# Supplementary material for: Research on the mechanical properties of hybrid Steel Fiber-Reinforced Self-Compacting Concrete based on the DIC method
Source: PLoS One. 2025 May 15;20(5):e0322450. doi: 10.1371/journal.pone.0322450 (PMC12080768; doi:10.1371/journal.pone.0322450)
Supplement: Attachment — Submitted filename: PONE-D-24-55030_R2_reviewer.pdf [file pone.0322450.s003.pdf]

PLOS ONE

The Influence of Hybrid Fiber on the Mechanical Properties of Self-Compacting Concrete

--Manuscript Draft--

**Manuscript Number:**	PONE-D-24-55030R2
**Article Type:**	Research Article
**Full Title:**	The Influence of Hybrid Fiber on the Mechanical Properties of Self-Compacting Concrete
**Short Title:**	The Influence of Hybrid Fiber on the Mechanical Properties of Self-Compacting Concrete
**Corresponding Author:**	Jianyi Gu<br>Chongqing Jiaotong University<br>Chongqing, CHINA
**Keywords:**	Self-compacting concrete; Blended steel fibers; mechanical properties; elastic modulus
**Abstract:**	The study investigates the impact of different volume fractions of mixed steel fibers on the me-chanical properties of self-compacting concrete. Preliminary tests were conducted on ordinary self-compacting concrete, single steel fiber concrete, and self-compacting concrete with 3% high volume mixed steel fibers. The results show that high volume mixed steel fiber self-compacting concrete specimens exhibit good bending performance, and the addition of steel fibers significantly inhibits the development of macro cracks, enhancing the concrete's resistance to cracking, effec-tively improving the brittleness of the concrete; when the fiber volume fraction is 1% 6mm+1% 13mm+1% 25mm, the compressive strength increased by 41.15%, and the flexural strength in-creased by 266.41%, and the elastic modulus reached 40.82MPa. The addition of high volume mixed steel fibers significantly improves the elastic modulus of self-compacting concrete, and the effect of mixed steel fibers is more pronounced.
**Order of Authors:**	Hang Hu
	Jianyi Gu
	Yang Tu
	Yi Zhou
	Fuwei Zhu
**Response to Reviewers:**	The authors would like to thank the reviewers for their suggestions. All tables have been revised, and all figures have been redesigned, with error bars or numerical values added to the corresponding data as indicated.
**Additional Information:**	
**Question**	**Response**
**Financial Disclosure**<br><br>Enter a financial disclosure statement that describes the sources of funding for the work included in this submission. Review the submission guidelines for detailed requirements. View published research articles from *PLOS ONE* for specific examples.	The author(s) received no specific funding for this work.

Powered by Editorial Manager® and ProduXion Manager® from Aries Systems Corporation

This statement is required for submission and **will appear in the published article** if the submission is accepted. Please make sure it is accurate.<br><br>**Funded studies**<br>Enter a statement with the following details:<br>• Initials of the authors who received each award<br>• Grant numbers awarded to each author<br>• The full name of each funder<br>• URL of each funder website<br>• Did the sponsors or funders play any role in the study design, data collection and analysis, decision to publish, or preparation of the manuscript?<br><br>Did you receive funding for this work?	
**Competing Interests**<br><br>Use the instructions below to enter a competing interest statement for this submission. On behalf of all authors, disclose any competing interests that could be perceived to bias this work—acknowledging all financial support and any other relevant financial or non-financial competing interests.<br><br>This statement is required for submission and **will appear in the published article** if the submission is accepted. Please make sure it is accurate and that any funding sources listed in your Funding Information later in the submission form are also declared in your Financial Disclosure statement.<br><br>View published research articles from *PLOS ONE* for specific examples.	The authors have declared that no competing interests exist.

**NO authors have competing interests**<br><br>Enter: *The authors have declared that no competing interests exist.*<br><br>**Authors with competing interests**<br><br>Enter competing interest details beginning with this statement:<br><br>*I have read the journal's policy and the authors of this manuscript have the following competing interests: [insert competing interests here]*<br><br>* typeset	
**Ethics Statement**<br><br>Enter an ethics statement for this submission. This statement is required if the study involved:<br><br>• Human participants<br>• Human specimens or tissue<br>• Vertebrate animals or cephalopods<br>• Vertebrate embryos or tissues<br>• Field research<br><br>Write "N/A" if the submission does not require an ethics statement.<br><br>General guidance is provided below. Consult the submission guidelines for detailed instructions. **Make sure that all information entered here is included in the Methods section of the manuscript.**	N/A

**Format for specific study types**<br><br>**Human Subject Research (involving human participants and/or tissue)**<br>• Give the name of the institutional review board or ethics committee that approved the study<br>• Include the approval number and/or a statement indicating approval of this research<br>• Indicate the form of consent obtained (written/oral) or the reason that consent was not obtained (e.g. the data were analyzed anonymously)<br><br>**Animal Research (involving vertebrate animals, embryos or tissues)**<br>• Provide the name of the Institutional Animal Care and Use Committee (IACUC) or other relevant ethics board that reviewed the study protocol, and indicate whether they approved this research or granted a formal waiver of ethical approval<br>• Include an approval number if one was obtained<br>• If the study involved *non-human primates*, add *additional details* about animal welfare and steps taken to ameliorate suffering<br>• If anesthesia, euthanasia, or any kind of animal sacrifice is part of the study, include briefly which substances and/or methods were applied<br><br>**Field Research**<br><br>Include the following details if this study involves the collection of plant, animal, or other materials from a natural setting:<br>• Field permit number<br>• Name of the institution or relevant body that granted permission	
**Data Availability**<br><br>Authors are required to make all data underlying the findings described fully available, without restriction, and from the time of publication. PLOS allows rare exceptions to address legal and ethical concerns. See the PLOS Data Policy and FAQ for detailed information.	No - some restrictions will apply

A Data Availability Statement describing where the data can be found is required at submission. Your answers to this question constitute the Data Availability Statement and **will be published in the article**, if accepted.<br><br>**Important:** Stating 'data available on request from the author' is not sufficient. If your data are only available upon request, select 'No' for the first question and explain your exceptional situation in the text box.<br><br>Do the authors confirm that all data underlying the findings described in their manuscript are fully available without restriction?	
**Describe where the data may be found in full sentences. If you are copying our sample text, replace any instances of XXX with the appropriate details.**<br><br>• If the data are **held or will be held in a public repository**, include URLs, accession numbers or DOIs. If this information will only be available after acceptance, indicate this by ticking the box below. For example: *All XXX files are available from the XXX database (accession number(s) XXX, XXX.).*<br>• If the data are all contained **within the manuscript and/or Supporting Information files**, enter the following: *All relevant data are within the manuscript and its Supporting Information files.*<br>• If neither of these applies but you are able to provide **details of access elsewhere**, with or without limitations, please do so. For example:<br><br>*Data cannot be shared publicly because of [XXX]. Data are available from the XXX Institutional Data Access / Ethics Committee (contact via XXX) for researchers who meet the criteria for access to confidential data.*<br><br>*The data underlying the results presented in the study are available from (include the name of the third party*	All relevant data are within the manuscript.

and contact information or URL).<br>• This text is appropriate if the data are owned by a third party and authors do not have permission to share the data.<br><br>* typeset	
Additional data availability information:	

Powered by Editorial Manager® and ProduXion Manager® from Aries Systems Corporation

Manuscript Click here to access/download;Manuscript;manuscript-V4.docx

The Influence of Hybrid Fiber on the Mechanical Properties of Self-Compacting Concrete

Hang Hu [1], Jianyi Gu [2,*] , Yang Tu [3] , Yi Zhou [3] and Fuwei Zhu [3]

1 Chengdu Communications Investment Group Co., Ltd, Chengdu, 610041, China; huhang750@hotmail.com

2 School of Civil Engineering, Chongqing Jiaotong University, Chongqing, 400074, China; gujianyi@163.com

3 Chengdu Road & Bridge Operation and Management Co., Ltd. Cheng Du, 610039, China; 18384228179@163.com, 13550333680@163.com , tiamozyp@outlook.com

* Correspondence: gujianyi@163.com ; +86 135-9402-5638

Abstract: The study investigates the impact of different volume fractions of mixed steel fibers on the mechanical properties of self-compacting concrete. Preliminary tests were conducted on ordinary self-compacting concrete, single steel fiber concrete, and self-compacting concrete with 3% high-volume mixed steel fibers. The results show that high-volume mixed steel fiber self-compacting concrete specimens exhibit good bending performance, and the addition of steel fibers significantly inhibits the development of macro cracks, enhancing the concrete's resistance to cracking, effectively improving the brittleness of the concrete; when the fiber volume fraction is 1% 6mm+1% 13mm+1% 25mm, the compressive strength increased by 41.15%, and the flexural strength increased by 266.41%, and the elastic modulus reached 40.82MPa. Adding high-volume mixed steel fibers significantly improves the elastic modulus of self-compacting concrete, and the effect of mixed steel fibers is more pronounced.

Keywords: Self-compacting concrete; Blended steel fibers; Mechanical properties; Elastic modulus

0. Introduction

One typical building material is concrete. Researchers are looking at the performance-enhancing advantages of adding steel fibers to concrete to increase its mechanical qualities. Determining the impact of steel fibers on enhancing concrete's performance after adding a specific quantity to the material has long been a popular area of study. Concrete's ductility and toughness can be enhanced by the inclusion of randomly distributed steel fibers, which also improves the concrete's resistance to impact and cracking. Steel fibers can help increase the resilience of concrete and resistance to freeze-thaw[1-2].

When steel fiber content rises, concrete's workability suffers from a number of issues, including easier agglomeration and decreased workability. The amount of conventional steel fibers in concrete is restricted at low doses, and the distribution of steel fibers will be impacted by vibration during

construction. The flexural and tensile properties of concrete can only be slightly improved by steel fibers. In actual applications, the content of steel fibers is typically between 0.5% and 2% due to poor fiber dispersion in concrete[3].

Numerous studies have demonstrated that self-compacting concrete's mechanical qualities and durability can be successfully increased by incorporating the proper quantity of single steel fibers into the material. Steel fibers with lengths of 33 mm and volume fractions of 0.1%, 0.2%, and 0.3% were tested to see how they affected the compressive strength of self-compacting concrete by M. Akbari et al[4]. The findings show that as the volume fraction rises, so does the compressive strength.

Athiyamaan V[5] mixed 0.25%, 0.50%, and 0.75% of hook-end micro-steel fibers with an aspect ratio of 60 with self-compacting concrete. The findings demonstrated that adding steel fibers to self-compacting concrete increased its flexural strength, inhibited the growth of cracks, avoided brittle failure, and enhanced strain performance.

Researchers[6] have found that adding a specific amount of steel fibers to self-compacting concrete can increase its strength (Aghaee et al., 2016). For steel fibers, a dosage of 0.75% is ideal.

Researchers like Mardani-Aghabaglou [7] discovered that the compressive strength of self-compacting concrete dropped by 6.3% and 9.5%, respectively, when the steel fiber percentage increased to 0.75 and 1.0%. This decline aligns with the self-compacting theory. It has to do with the concrete losing its capacity to self-compact due to a decline in the workability of the concrete mixture itself, which lowers the effectiveness of self-compacting concrete.

For the purpose of to conduct comparative studies, researchers like Saba et al. [8] discovered that after adding 0~15% silica fume to self-compacting concrete, they added steel fibers with a volume content of 0~1% and an aspect ratio of 65~80. The test findings indicate that self-compacting concrete is significantly strengthened and toughened by steel fiber with an aspect ratio of 85 when the silica fume content is 15%, the volume content is 1%, and these conditions hold.

Researchers Sun et al. [9] discovered that the performance of self-compacting concrete is affected in a number of ways by the addition of steel fibers. They added steel fibers to self-compacting concrete in experimental trials at varying volumetric doses. The findings demonstrated that adding steel fibers by volume to self-compacting concrete will The tensile and flexural strengths of self-compacting concrete rise by 70% when the amount is increased from 0.5% to 1.25%, but the compressive strength reduces by 11%. It demonstrates that adding steel fiber greatly increases the tensile and flexural strength of self-compacting concrete while inhibiting its compressive strength.

Ma et al. [10] discovered that the compressive strength of self-compacting concrete could be raised by 19%–42% by adding steel fibers with a volume content of 0~2%, a length of 13 mm, and an aspect ratio of 65.

When choosing steel fibers for hybrid fiber-reinforced self-compacting concrete, two common varieties are considered. One is regular steel fiber, which has an aspect ratio of 40 to 65, a length that typically ranges from 15 to 35 mm, and a diameter that is mostly concentrated at 0.5 mm. Regular steel fiber is the other. It is a steel fiber that is extremely short and fine. Its shorter length makes it easier to mix uniformly than regular steel fiber and won't significantly impair concrete's performance. Different methods of mixing two kinds of steel fibers are used to enhance the mechanical characteristics of self-compacting concrete.

At present, most research focuses on improving the mechanical properties of self-compacting concrete after mixing two steel fibers with different aspect ratios. Burcu Akcay[11] et al. artificially studied the effects of steel fibers with different aspect ratios on the performance of self-compacting concrete, Add 1% straight steel fiber in volume fraction to self-compacting concrete (Length 6mm, diameter 0.15mm) and 0.5% hook-end steel fiber (length 30mm, diameter 0.55mm), The results show that these fibers can be dispersed evenly in self-compacting concrete without clumping, thus improving the toughness of the concrete. Deeb[12] et al. added 5% steel fiber (Length 6mm, diameter 0.16mm) and 1% steel fiber (Length 13mm, diameter 0.16mm) by volume to self-compacting concrete, experimental studies were conducted on the fluidity of concrete. The results prove that ultra-short ultra-fine steel fibers mixed with a higher content can produce self-compacting concrete with fluidity that meets the requirements. However, the article does not describe the enhanced effect of hybrid steel fibers on the properties of self-compacting concrete. Dimas[13-15] [illegible] al. used 12mm straight-end steel fiber (diameter is 0.18mm) with a volume content of 0.5% and 1.5% 35mm hook-end steel fiber (diameter 0.55mm) as reinforcing fibers. The mechanical properties such as fracture and bending resistance of self-compacting concrete were studied through compression, tension, and bending tests. Experimental results show that the normal serviceability limit state of self-compacting concrete has been improved.

Scholarly research based on the aforementioned data indicates that little is known about the hybrid impacts of three distinct aspect ratio steel fibers at this time. On the effect of hybrid reinforced self-

compacting concrete on performance, more investigation and analysis can be carried out. Drawing from prior studies, this investigation aimed to examine the effects of adding steel fibers with varying aspect ratios to self-compacting concrete at a volume content of 3% on the concrete's properties. To achieve self-compacting concrete, this work conducts experimental research on the fundamental mechanical properties of 3% hybrid steel fibers. The result is a high-content, self-compacting steel fiber concrete that performs exceptionally well throughout construction.

1. Materials and methods

1.1 Test materials and proportions

（1）Cement.The cement is P·O42.5 ordinary Portland cement produced by Chongqing Lafa base's dimensional cement formula. Its physical and mechanical properties are shown in Table 1.

Table1　Basic physical and mechanical properties of cement

Apparent density/（g • cm-3）	Specific surface area/ (m2 • kg-1)	Sieve residual ratio(45μm sieve, by mass)/%	Water demand ratio for normal consistency (by mass)/%	Setting time/min		3d strength/MPa	
				Initial	Final	Compressive	Flexural
3.1	345	8.4	26.80	165	226	30.0	5.90

（2）Fly ash. Fly ash is low calcium Class F fly ash produced and provided by Chongqing Hualuo Fly Ash Co., Ltd.

Table 2 Physical properties of fly ash

Apparent density/ g • cm-3）	Sieve residual ratio(45 μm sieve, by mass)/%	IL(by mass)/%	Water demand ratio(by mass)/%	Activity index/%	
				7d	28d
2300	0	5	95	83	95

（3）Aggregate. The fine aggregate is limestone machine-made sand with a fineness modulus of 3.2, a fine powder content of 3%, an apparent density of 2712kg/m³, and a bulk density of 1521 kg/m³, Coarse aggregate is 5~10mm crushed stone, with an apparent density of 2.78g/cm³ and a bulk density of 1.45g/cm³.

Table 3 Gradation of fine aggregate

Sieve diameter/mm	Sieve residual ratio(by mass)/%	Cumulati[illegible]ieve residual ratio(by mass)/%	Di/mm
4.75	0	0	0
2.36	19.3	19.3	3.35
1.18	17.4	36.7	1.67
0.60	22.1	58.8	0.84
0.30	21.7	80.5	0.42

0.15	13.7	94.2	0.21
0.075	3.6	97.8	0.11
0	2.2	100	0.03

Table 4 Gradation of coarse aggregate

Sieve diameter/mm	Sieve residual ratio(by mass)/%	Cumulative sieve residual ratio(by mass)/%	Di/mm
16	0	0	0
13.2	0.27	0.27	14.53
9.5	0.40	0.67	11.20
4.75	0.33	1.00	6.74

（4）Steel fiber. The main performance is shown in Table 5.

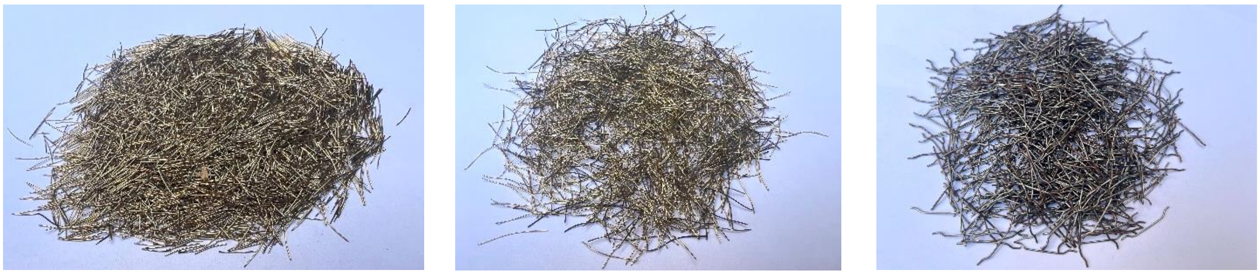

6mm 13mm 25mm

Figure 1 Steel fibers with different aspect ratios

Table 5 Types of steel fibers and main physical and mechanical properties

Steel fiber types	Length /mm	Aspect ratio	Density（t/m³）	Elastic Modulus（GPa）	Tensile strength（MPa）
Ordinary steel fiber (hook-end)	25	33	7.8	210	850
Ordinary steel fiber	13	65	7.8	240	＞850
Ultra-short ultra-fine steel fiber	6	35	7.8	240	＞850

（5）Water-reducing agent. Water reducing agent (WR) is a high-efficiency polycarboxylate water-reducing agent mother liquor with a solid content (mass fraction) of 50% to 60% and should be diluted 6.5 times.

（6）Water. Using tap water from the laboratory of Chongqing Jiaotong University.

（7）Mix ratio selection. The mix ratio of this test was carried out based on the mix ratio determined in the previous test study. Table 6 records the mix ratio of steel fiber self-compacting concrete with different steel fiber volume content of 1m³.

Table 6 Mix ratio of steel fiber self-compacting concrete specimens (1m³/kg)

Number	Cement（kg）	Water（kg）	Fly ash（kg）	Coarse aggregate（kg）	Fine aggregate（kg）	Water reducing agent（%）	Steel fiber content（%）
1	1	0.37	0.43	1.37	1.06	0.16%	0
2	1	0.37	0.43	1.37	1.06	0.16%	3%6mm
3	1	0.37	0.43	1.37	1.06	0.16%	3%13mm

4	1	0.37	0.43	1.37	1.06	0.16%	3%25mm
5	1	0.37	0.43	1.37	1.06	0.16%	1%6mm+1%13mm+1%25mm

(For the convenience of research, 3%6 represents 6mm steel fiber with a volume content of 3%, and other naming methods are the same)

Self-compacting steel fiber concrete should use a mandatory mixer. In this experiment, coarse aggregate, fine aggregate, and cement are first poured in, and then the steel fibers are slowly added in batches. The water mixed with the water-reducing agent is poured into the mixer. After the addition is completed, continue stirring for one minute. When there is a large amount of fiber or fiber, a fiber disperser can be used.

1.2 Experiment preparation

Before the test, the self-compacting concrete fluidity test and passing ability test were carried out [16]. The slump expansion diameter and J-ring expansion of self-compacting concrete with different volume dosages are shown in Figure 2 and Figure 3.

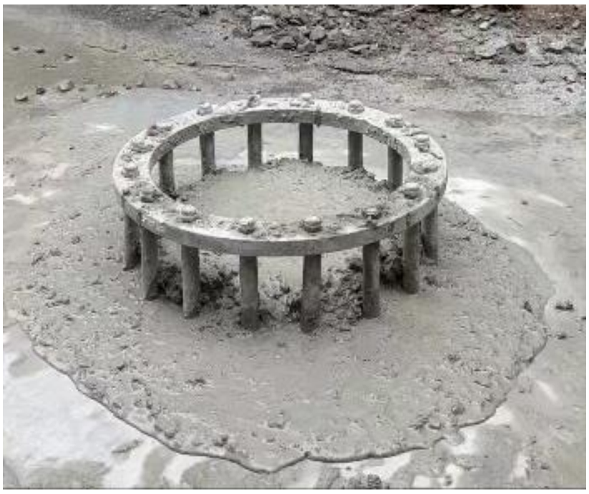

Figure 2 Slump expansion of steel fiber self-compacting concrete

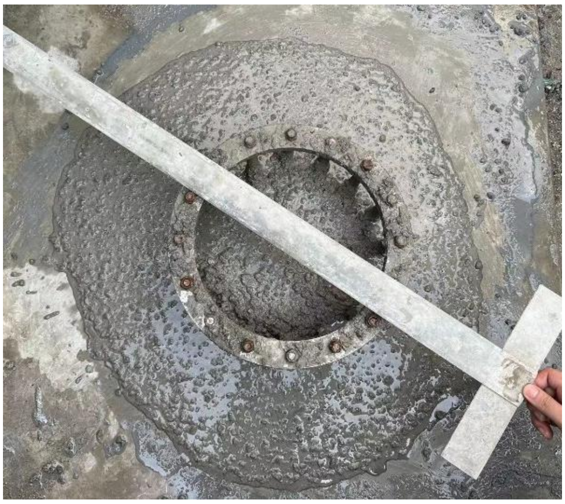

Figure 3 J-ring expansion of steel fiber self-compacting concrete

The experimental data are shown in Table 7, The experimental data are shown in Table 7, according to the "Technical Specification for Application of Self-Compacting Concrete" JGJ/T 283-2012, the standard for the slump flow of self-compacting concrete is 500mm to 800mm.

Table 7 Slump expansion diameter and J-ring expansion of steel fiber self-compacting concrete under different steel fiber content

Number	Length	Fiber content	slump propagation （mm）	J ring expansion （mm）
S-0	0	0	840	800
3%6	6mm	3%6mm	660	500

3%13	13mm	3%13mm	520	410
3%25	25mm	3%25mm	730	610
6+13+25	6mm+13mm+25mm	1%6mm+1%13mm+1%25mm	650	530

The target value for the slump flow of self-compacting concrete should not be less than 500mm; therefore, the self-compacting concrete with various steel fibers meets the construction requirements.

1.3 Experimental method

In this study, the mechanical properties of self-compacting concrete were characterized by compressive strength, flexural strength, and elastic modulus, so cube specimens and beam specimens were prepared for corresponding tests. This experiment conducted a preliminary experimental study on five groups of specimens: self-compacting concrete, single-content steel fiber concrete, and self-compacting concrete mixed with ordinary steel fiber and hook-end steel fiber at 3% content. Comparative analysis of the effect of steel fibers on the properties of self-compacting concrete. The number of test specimens for each group of solutions is 30 cube compressive strength test, flexural test, and bending and tensile test. The total number of specimens is 90. The size and quantity of specimens are summarized in Table 8.

Table 8 Dimensions and quantity of specimens

Test content	Specimen size（mm）	Number of test pieces	Number of samp[illegible]
Compressive strength	100×100×100	6	30
Flexural strength	100×100×400	6	30
Elastic Modulus	100×100×400	6	30

1.3.1 Cube compressive strength test

The primary indicators for evaluating the strength of concrete materials are compressive strength and flexural strength[17]. Use a cube test block with a size of 100mm×100mm×100mm, Prepare three identical specimens in each group, remove the mold after 24 hours of molding, After curing the specimen in the curing box for 28 days, and clean the surface of the specimen. Place the axis of the specimen perpendicularly to the center of the lower plate, and adjust the upper-pressure plate to be close to but not touching the upper surface of the test piece. Start the testing machine to preload. The loading speed during preloading is 0.5MPa/s, perform physical alignment adjustments and pressure stability adjustments. After the preloading is completed, the specimen is loaded at a constant speed of 2KN/s until the specimen is damaged, and then the load is slowly unloaded.

The compressive strength results of the test block should be calculated according to the following formula：

$$f_{cc} = \frac{F}{A} \qquad (1.1)$$

In the formula：

f_{cc}—Steel fiber concrete cube compressive strength（MPa）;

F—Ultimate load（N）;

A—Average value of the upper and lower pressure areas of the test block（mm^2）。

1.3.2 Flexural strength test

According to the Chinese National Standard GB/T 50081—2002 "Standard for Testing Methods of Mechanical Properties of Ordinary Concrete." The size of the self-compacting concrete steel fiber test block is 100mm×100mm×400mm. The test was conducted on a hydraulic universal testing machine. The long axis of the test block is perpendicular to the support axis. Load at a constant speed of 50N/s until the test block is destroyed. After the specimen is cured in the curing box for 28 days, and the surface and inspected the surface of the specimen, Place the specimen on the testing machine with its long axis perpendicular to the support cylinder. The distance between the two cylindrical supports below is 0.3 meters, and constraints adopt fixed hinges and universal rolling hinge supports. Before starting the testing machine, first preload the test block with a loading force of 1kN. Check whether the test block is placed flat to ensure that the test is carried out in a standardized and safe manner. Before the initial failure of the test block occurs, the loading rate is 0.06MPa/s. Once cracks appear in the test block, the loading method is changed, displacement control is used, the load is loaded until the test piece is damaged, and then the load is slowly unloaded. The loading process is shown in Figure 4.

The flexural strength of the test block is expressed in MPa and is calculated according to the following formula:

$$f_r = \frac{3FL}{2bh^2} \qquad (1.2)$$

In the formula:

f_r—Steel fiber concrete flexural strength, MPa,

P—Destruction load applied to the middle part of the specimen when breaking (N),

L—Support spacing (mm),

b—Specimen height (mm),

h—Specimen width (mm).

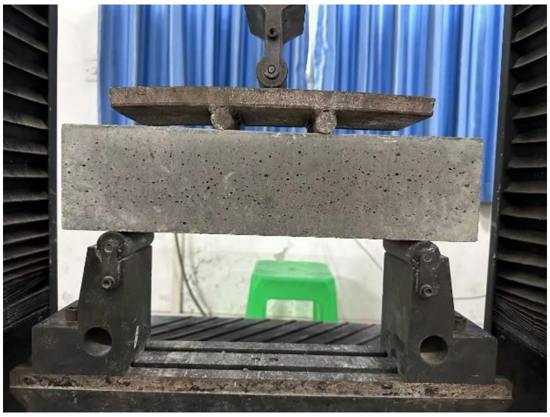

Figure 4 Flexural test loading of self-compacting steel fiber concrete

1.3.3 Elastic modulus test

According to GB/T 50081-2002 "Standard for Test Methods of Mechanical Properties of Normal Concrete", the specimen is a prism with edge lengths of 150mm × 150mm × 300mm. Attach the strain gauge to the specimen whose side hole diameter is less than 5mm and depth is less than 2mm. In order to consider the error factor of uneven distribution of aggregates, use a smear rod to stick to the strain gauge at the center of the left and right sides of the specimen, and the center lines of both sides need to coincide. After the smeared area is dry, place the specimen on the pressure-bearing platform according to the specifications and prepare to add load on the universal testing machine. Take 1/2 of the average value of the bending and tensile ultimate load as the load standard F0.5 for the bending and tensile elastic modulus test, and perform 5 loading and unloading cycles, starting from 10KN and loading at a speed of 0.2mm/min.

The flexural and tensile elastic modulus of concrete should be calculated as follows:

$$E_f = \frac{23L^3(F_{0.5}-F_0)}{1296J|\Delta_{0.5}-\Delta_0|} \quad (1.4)$$

In the formula:

E_f—Concrete bending and tensile elastic modulus (MPa),

$F_{0.5}$、F_0—Final load and initial load (N),

$\Delta_{0.5}$、Δ_0—Corresponding to the dial indicator reading of $F_{0.5}$ and F_0 (mm);

L—Distance between specimen supports (L=450mm)；

J—Moment of inertia of specimen section, $J = \frac{1}{12}bh^3$ (mm4).

2. Results and analysis

2.1 Cube compressive strength

Figure 5 shows the compression failure modes of ordinary concrete, single dosage, and hybrid steel fiber concrete cubes.

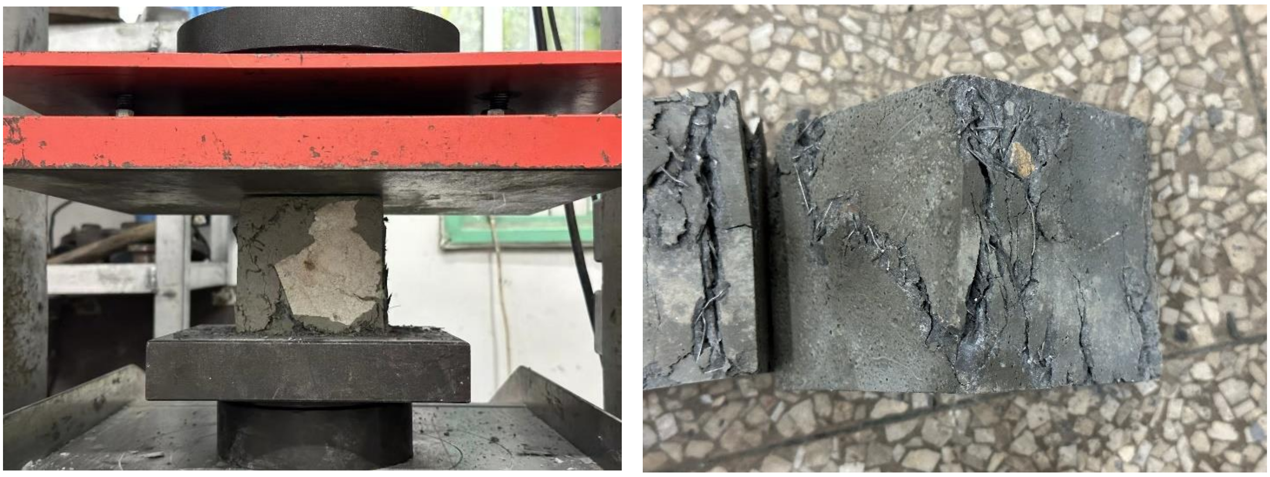

Figure 5 Compression failure pattern of concr[illegible]ube specimens

According to the compression failure pattern of the concrete specimen in Figure 2-1, it can be seen that when the self-compacting concrete specimen is mixed with steel fibers, its peeling speed slows down under compressive load, the time it continues to bear pressure after peeling increases, and the peeling is more uniform.

According to the test, the data shown in Table 9 is obtained. The data in the table shows that the addition of steel fibers can effectively improve the compressive strength of self-compacting concrete, Compared with ordinary self-compacting concrete, when the fiber content is 3%, the increase in the compressive strength of concrete with steel fibers with different aspect ratios is not much different, Among them, the addition of 13mm steel fiber greatly improves the compressive strength of self-compacting concrete. By mixing three types of steel fibers into self-compacting concrete, the compressive strength of the concrete has been significantly improved. Compared with ordinary self-compacting concrete, the compressive strength has increased by 41.15%. At the same time, when the volume fraction of steel fibers is the same, the compressive strength of hybrid steel fiber concrete is increased by about 20% compared with steel fiber alone. But overall, the addition of steel fibers has a limited effect on enhancing the compressive strength of self-compacting concrete.

Table 9 Cube compressive strength test results (28d, MPa)

Number	Fiber content	Compressive strength (MPa)	Improvement
S-0	0	73.65	—
3%6	3%6mm	82.56	12.09%
3%13	3%13mm	85.28	15.79%
3%25	3%25mm	83.26	13.05%

6+13+25	1%6mm+1%13mm+1%25mm	103.96	41.15%

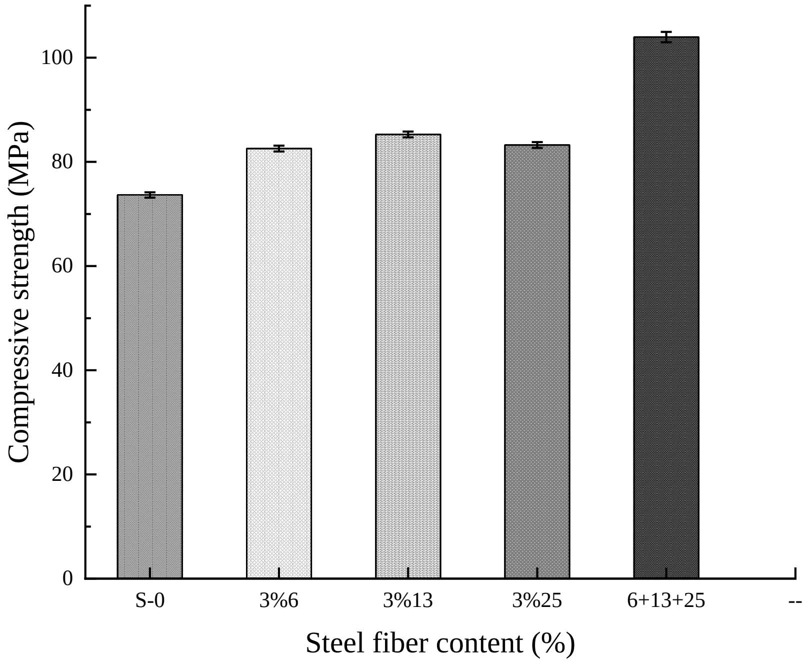

Figure 6 The relationship between the compressive strength value of self-compacting steel fiber concrete cube and the volume content of steel fiber

2.2 Flexural strength

Figure 7 shows the flexural failure morphology of ordinary concrete, single dosage, and hybrid steel fiber concrete cubes.

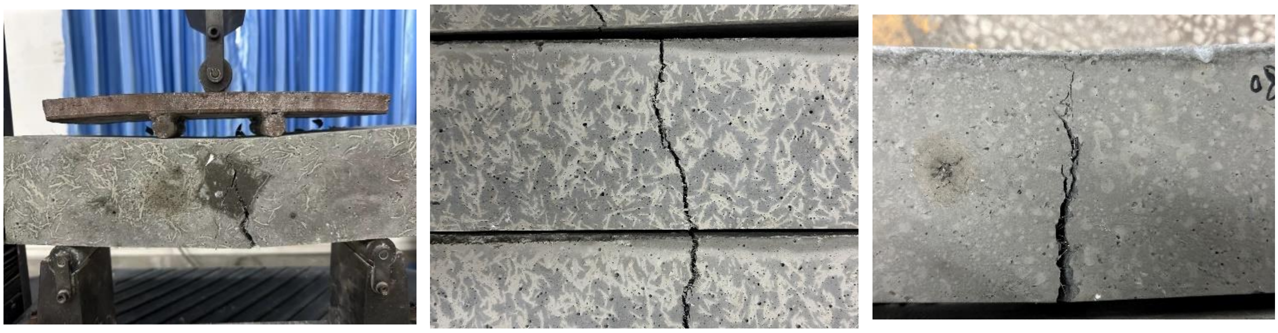

Figure 7 Concrete flexural failure pattern

It can be seen from the failure shape that under the ultimate load, cracks soon appeared at the edge of the tensile zone at the bottom of the concrete specimen without adding steel fibers. Under the influence of stress concentration and tip effect, the crack immediately expanded to the top, and a clear crack appeared. At the same time, several concrete blocks fell off in the tension area, accompanied by a crisp and loud breaking sound, plain concrete blocks quickly failed and stopped working. The flexural concrete test block with steel fiber added will have a slower time of cracking than the plain concrete test block. Under the action of concentrated force, a main crack first appears in the bottom tension zone. As the load increases, the main crack gradually expands from the bottom of the tension zone to the top of the beam until the specimen is completely cracked. The flexural strength obtained

through the test is shown in Table 10.

Table 10 Flexural strength test results (28d, MPa)

Number	Fiber content	Flexural strength (MPa)	Improvement
S-0	0	3.93	—
3%6	3%6mm	8.41	113.95%
3%13	3%13mm	14.96	280.66%
3%25	3%25mm	12.24	211.45%
6+13+25	1%6mm+1%13mm+1%25mm	14.47	268.19%

As can be seen from Figure 8, steel fibers can effectively improve the flexural strength of self-compacting concrete. Compared with ordinary self-compacting concrete, the flexural strength of self-compacting concrete mixed with fibers has a greater increase. When the volume content is 3%, the 13mm steel fiber has the most obvious effect on enhancing the flexural strength of self-compacting concrete, while the addition of ultra-short and ultra-fine 6mm steel fibers has no significant effect on the flexural strength of self-compacting concrete. This shows that as the aspect ratio of steel fibers increases, the flexural strength of self-compacting concrete continues to increase. After mixing three steel fibers with different aspect ratios, mixing 1%6mm+1%13mm+1%25mm into self-compacting concrete, it can be seen that the reinforcing effect of mixed fibers on self-compacting concrete is not as good as the reinforcing effect of 13mm steel fiber alone. Analyze the reasons: Steel fibers with different aspect ratios within the concrete are not easily dispersed fully and evenly, and the agglomeration of the fibers with cement slurry or aggregate increases the internal pores of the specimen, resulting in a limitation in the enhancement effect of mixed fibers on the flexural strength of self-compacting concrete.

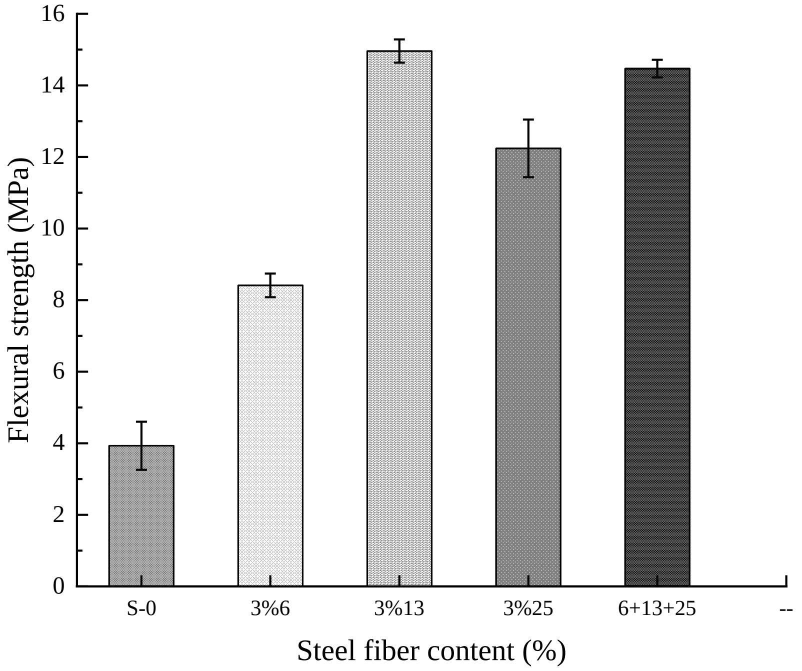

Figure 8 The relationship between the flexural and tensile elastic m[illegible]dulus of self-compacting steel fiber concrete and the volumetric content of ste[illegible]iber

2.3 Elastic Modulus

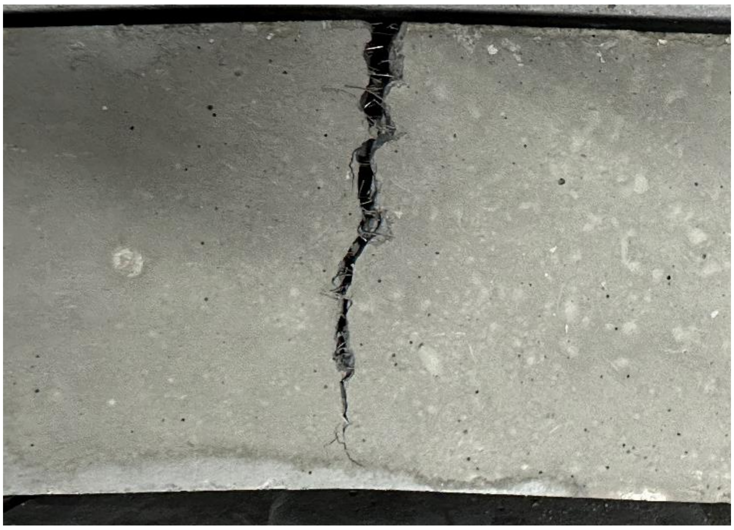

Figure 9 Elastic Modulus test loading of self-compacting steel fiber concrete

According to the test results, it can be seen that ordinary self-compacting concrete specimens have very long cracks after being loaded. Basically running through the entire rectangular parallelepiped, the crack develops from top to bottom from the upper platform, and the width of the crack is very wide when it is close to the upper platform, and is narrower near the lower platform. The cracks in the mixed steel fiber specimens have a narrower width, develop slower, and can withstand the load for a longer time. The elastic modulus obtained through the test is shown in Table

11.

Table 11 Elastic modulus test results (28d, MPa)

Number	Initial load $F_0(kN)$	Test piece pressure area $A(mm^2)$	Average elastic modulus $E_c(MPa)$
S-0	10.0	10000	26490
3%6	10.0	10000	34880
3%13	10.0	10000	35230
3%25	10.0	10000	32820
6+13+25	10.0	10000	40820

It can be seen from Figure 10 that when steel fibers are added, the elastic modulus of self-compacting concrete is significantly improved. At the same dosage, steel fibers with lengths of 6 mm and 13 mm improve the elastic modulus of self-compacting concrete more than the addition of 25 mm steel fibers, indicating that the performance improvement of self-compacting concrete by hook-end steel fibers is weak. It can be clearly seen from the figure that hybrid steel fibers have a more obvious increasing trend in the elastic modulus of self-compacting concrete. This shows that the incorporation of hybrid steel fibers has a certain effect on improving the elastic modulus, but generally speaking, steel fibers have little effect on the elastic modulus.

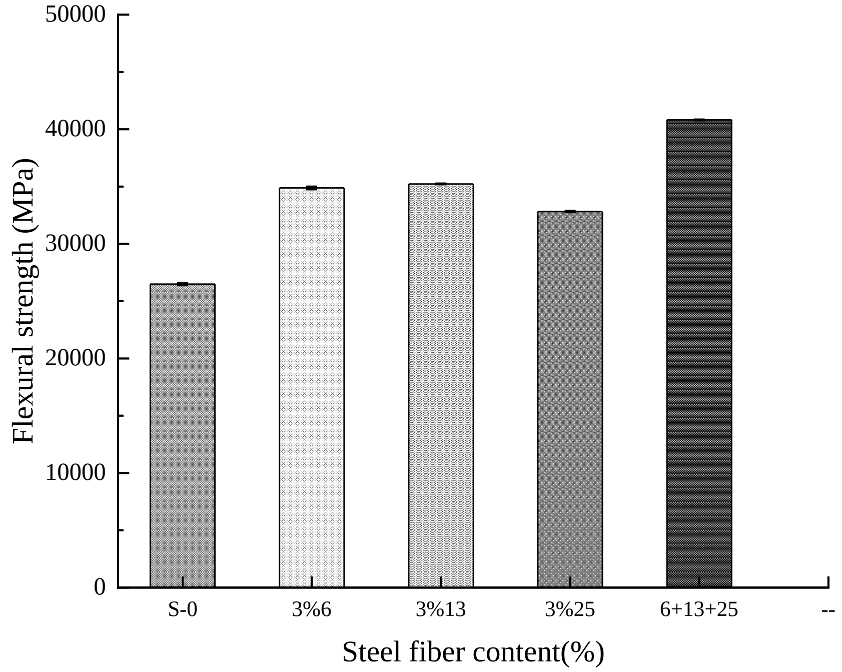

Figure 9 The relationship between the elastic modulus of self-compacting steel fiber concrete and the volume content of steel fiber

2.4 Tension-compression ratio

In order to study the impact of steel fiber on the strength of concrete, this article further analyzed the test data conducted previously to obtain the tension-to-compression ratio of the specimen and plotted it with the steel fiber content as the X-axis and the tension-to-compression ratio as the Y-axis. As shown in Figure 11, The Y-axis data is the ratio of the flexural strength of the specimen to the compressive strength of the specimen.

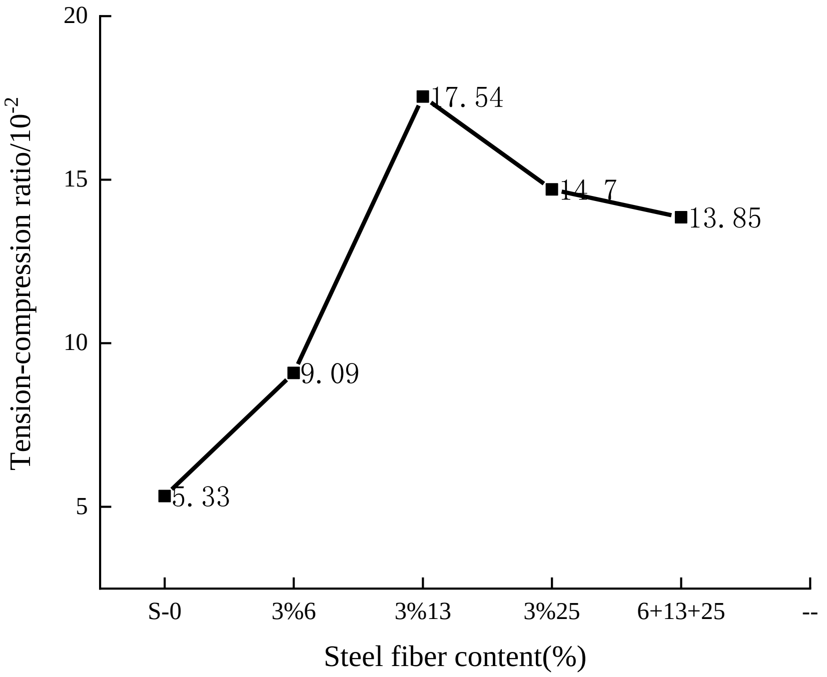

Figure 11 The relationship between the tension-compression ratio of self-compacting steel fiber concrete and the volume content of steel fiber

The influences of steel fibers with different lengths and contents on the tension-compression ratio of concrete are diverse. Among them, the 13mm steel fibers with a volume content of 3% can enhance the tension-compression ratio of concrete to the greatest extent. While the effect of mixing steel fibers of multiple lengths is relatively weaker, it is still better than the situation without steel fibers and some cases of single-length steel fibers. Consequently, steel fibers can effectively improve the tension-compression ratio of self-compacting concrete.

3. Discussion

3.1 Optimal dosage

According to the compression, flexural, and elastic modulus test results, it can be seen that the addition of steel fibers can effectively improve the mechanical properties of self-compacting concrete. Since the flexural strength of 13mm steel fiber alone increases significantly, it is difficult to determine the specific mixing ratio that will have the most obvious improvement effect on self-compacting

concrete.

In order to find the optimal blending ratio, data normalization is used to narrow the selection criteria to between 0 and 1. By accumulating the regularization criterion values, the comprehensive impact of steel fibers on self-compacting concrete is determined.

The data normalization formula is as follows,

$$D_n = \frac{D - D_{min}}{D_{max} - D_{min}} \quad (3\text{-}1)$$

In the formula:

D--- the original value of the test data,

D_{max} --- the maximum value of the test data,

D_{min} --- the minimum value of the test data,

D_n ---the normalized value of the test data.

In this study, the experimental data obtained above were imported into the Origin software, and the comprehensive impact of steel fibers on self-compacting concrete was obtained through normalized calculations, as shown in Figure 12. It can be seen from the normalized data that when the fiber content is 1%6mm+1%13mm+1%25mm, the improvement effect on the mechanical properties of self-compacting concrete is the best. It can be seen that hybrid steel fibers can effectively inhibit the development of concrete cracks and improve the cracking resistance of self-compacting concrete.

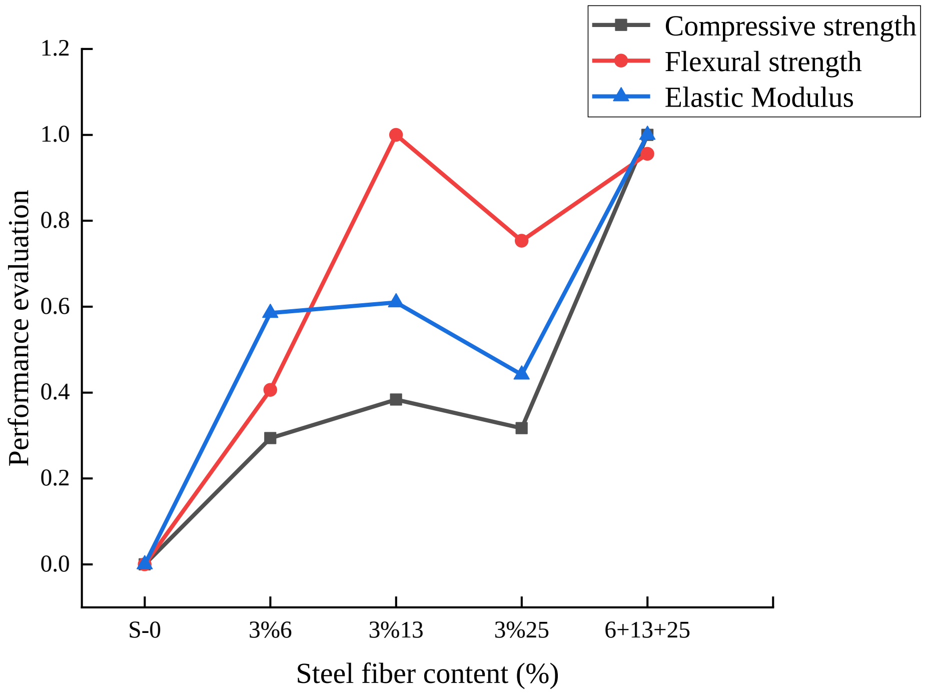

Figure 12 Comprehensive effect of fiber content on self-compacting concrete

3.2 Comparison

To prove the reliability of this study, this article selects three similar studies from previous years for comparative analysis. In 2019, researchers such as Alabduljabbar [19] added steel fibers with volume fractions of 0, 1%, 1.5%, and 2% to self-compacting concrete respectively. Research results show that when the volume content is 2%, the compressive strength and tensile strength of steel fiber reinforced self-compacting concrete reach the peak. Ghorbani (2020)[20] added steel fibers with a volumetric content of 0~1.65% to self-compacting concrete for research. The results showed that the performance of self-compacting concrete is optimal when the content is 1.65%. Abbas (2021)[21] also added steel fibers with a volume content of 0~2% in self-compacting concrete, the test results show that when the dosage is 2%, the self-compacting concrete has the highest strength. Based on the above three test results, this study comparatively analyzes the reinforcing effects of steel fibers at different dosages.

Table 12 Comparison of experimental data

**R[illegible]**	**Steel fiber percentages(%)**	**Days**	**Optimum(%)**	**Compression strength (MPa)**	**Flexure strength (MPa)**
Alabduljabbar et al.,(2019)	0~2	28	2	+17.2%	+1.96%
Ghorbani et al.,(2020)	0~1.65	28	1.65	-1.51%	+28.57%
Abbas et al.,(2021)	0~2	28	2	+40.0%	-
This Research	0~3	28	3	+41.15%	268.19%

It can be seen from the growth data listed in Table 12 that different steel fiber content has different reinforcing effects on self-compacting concrete. Compared with other experimental research results, the mechanical properties of steel fiber reinforced self-compacting concrete obtained in this study have obvious hybrid effects. This study explains the reinforcement mechanism of self-compacting concrete by hybrid steel fibers with different aspect ratios. It can be seen from the data in the table that this research shows strong performance advantages in both compressive strength and flexural strength. Higher compressive strength means that the material is less likely to be damaged when subjected to pressure and can be applied to scenarios with higher requirements for bearing capacity. The extremely high flexural strength indicates that the material has better toughness and stability when subjected to bending forces, and is suitable for structures or components that need to resist bending deformation. This makes the research results potentially have wider applicability and competitiveness in practical applications.

4. Conclusion

（1）When mixed steel fibers with a volume content of 1% 6mm + 1% 13mm + 1% 25mm are added to the concrete, the compressive strength of self-compacting concrete increases by 41.15%; the flexural strength increases by 268.19% respectively. Therefore, the compressive and flexural strength of self-compacting concrete can be improved by incorporating hybrid steel fibers.

（2）Steel fibers can significantly improve the damage state of concrete. When a steel fiber concrete specimen fails, the cracks usually do not penetrate the entire specimen like ordinary concrete. The test block generally does not break into two pieces and has an obvious initial cracking load. The test block generally cracks without breaking, and its failure shows a certain ductility.

（3）When steel fibers are added to the concrete, the test block shows obvious ductility during the loading process, which is manifested as continuous cracking of the test piece.

It can be seen that the basic mechanical properties of the self-compacting hybrid steel fiber concrete obtained by mixing three different aspect ratio steel fibers meet the requirements of self-compacting concrete as the fiber volume content increases. It not only achieves self-compacting properties that are difficult to achieve at high dosages, but also meets the workability of the mixture and complies with national standards. Secondly, after multiple experimental tests, the self-compacting hybrid steel fiber concrete test block has good compressive and flexural properties. Moreover, steel fibers can significantly inhibit the development of macro cracks, making concrete more resistant to cracking.

References:

[1] Zhino A. Shawais,Khamees N. Abdulhaleem,Saleh H. Ahmed,Hussein M. Hamada,Van R. Mohammed.Influence of Recycled Coarse Aggregate and Steel Fiber on the Workability and Strength of Self-Compacting Concrete[J].IOP Conference Series: Earth and Environmental Science,2024,1374(1):012084-012084.

[2] Nabeel A.Farhan,M.Neaz Sheikh,Muhammad N.S.Hadi.Effect of Steel Fiber on Engineering Properties of Geopolymer Concrete[J].ACI Material Journal,2020.5.

[3] Alberto José Palacio,Bárbara Belén Raggiotti,Viviana Carolina Rougier.Development and experimental verification of a design method for steel fibre-reinforced high strength lightweight self-compacting concrete[J].Construction and Building Materials,2024,453139012-139012.

[4] Mohamed M. Al Rifai,Karol S. Sikora,Muhammad N.S. Hadi.Effect of micro steel fibers volume fraction on behavior of high-strength self-compacting concrete[J].Construction and Building Materials,2024,450138709-138709.

[5] Athiyamaan V, G. Mohan Ganesh1. Analysis of the Alignment of Micro-Steel Fibers in Admixture-Based Self-Compacting Concrete (MSFR-SCC) using NDT and Evaluation of Its Effect on the Modulus of Rupture [J]. International Journal of Technology. 10(1): 5-15 2019.

[6] Aghaee, K., Yazdi, M. A., & Tsavdaridis, K. D. Investigation into the mechanical properties of structural lightweight concrete reinforced with waste steel wires. Magazine of Concrete Research, 67(4), 197–205.2015.

[7] Mardani-Aghabaglou, A., Tuyan, M., Yılmaz, G., Arıöz, Ö., & Ramyar, K. Effect of different types of superplasticizer on fresh, rheological and strength properties of self-consolidating concrete. Construction and Building Materials, 47, 1020–1025.2013.

[8] Saba, A. M., Khan, A. H., Akhtar, M. N., Khan, N. A., Koloor, S. S. R., Petru, M.,& Radwan, N. Strength and flexural behavior of steel fber and silica fume incorporated self-compacting concrete. Journal of Materials Research and Technology, 12,1380-1390.2021.

[9] Sun, L., Wenju, Hu., Yuan,Y, Cao, X., & Lei, Bo. Dynamic performance of the shading-type building-integrated photovoltaic claddings. Procedia Engineering, 121,930-937.2015.

[10] Ma, H. L., Cui, C., Li, X., & Hu, S. L. Study on mechanical properties of steel fiber reinforced autoclaved lightweight shell-aggregate concrete.Materials and Design, 1980–2015(52), 565–571.2013.

[11] Akcay,Burcu;Tasdemir, Mehmet Ali.Mechanical behaviour and fibre dispersion of hybrid steel fibre reinforced self-compacting concrete.[J].Construction & Building Materials.2012,Vol.28(No.1)：287-293.

[12] Deeb R.Development of self-compacting high and ultra high performance concretes with and without steel fibres[J].Cement&Concrete Composites,2012,34:185-190.

[13] Dimas Alan Strauss Rambo,Flávio de Andrade Silva,Romildo Dias Toledo Filho.Effect of steel fiber hybridization on the fracture behavior of self-consolidating concretes[J] Cement & Concrete Composites.2014.

[14] Dimas Alan Strauss Rambo,Flávio de Andrade Silva,Romildo Dias Toledo Filho.Flexural behavior of hybrid steel fiber reinforced self-consolidating concretes[J] Civil Engineering. 67(1), 27-32, jan. mar. 2014.

[15] Dimas Alan Strauss Rambo,Flávio de Andrade Silva,Romildo Dias Toledo Filho.Mechanical behavior of hybrid steel-fiber self-consolidating concrete: Materials and structural aspects[J] Materials and Design.2014.

[16] ASTM C 1621. Standard test method for passing ability of self-compacting concrete by J ring.[S] ASTM Standards; 2006.

[17] Iqbal, S., Ali, A., Holschemacher, K., & Bier, T. A. Effect of change in micro steel fiber content on properties of high strength steel fiber reinforced lightweight self-compacting concrete (HSLSCC). Procedia Engineering, 122, 88-94, 2023.

[18] RILEM 50-FMC. Determination of the fracture energy of mortar and concrete by means of three-point bend tests on notched beams.[J] Materials and Structures 1985; 18(4):285–90.

[19] Alabduljabbar, H., Alyousef, R., Alrshoudi, F., Alaskar, A., Fathi, A., & Mohamed,A. M. Mechanical effect of steel fiber on the cement replacement materials of self-compacting concrete. Fibers, 7(4), 36.2019.

[20] Ghorbani, S., Sharif, S., Rokhsarpour, H., Shoja, S., Gholizadeh, M., Rahmatabad,M. A. D., & de Brito, J. Effect of magnetized mixing water on the fresh and hardened state properties of steel fibre reinforced self-com- pacting concrete. Construction and Building Materials,248, 118660.2020.

[21] Abbas, S. A., Ali, 1. F., & Abdulridha, A.A. Behavior and strength of steel fiber reinforced self-compacting concrete columns wrapped by carbon fiber reinforced polymers strips. International Journal of Engineering, 34(2),382-392.2021.

The Influence of Hybrid Fiber on the Mechanical Properties of Self-Compacting Concrete

Hang Hu [1], Jianyi Gu [2,*] , Yang Tu [3] , Yi Zhou [3] and Fuwei Zhu [3]

1 Chengdu Communications Investment Group Co., Ltd, Chengdu, 610041, China; huhang750@hotmail.com

2 School of Civil Engineering, Chongqing Jiaotong University, Chongqing, 400074, China; gujianyi@163.com

3 Chengdu Road & Bridge Operation and Management Co., Ltd. Cheng Du, 610039, China; 18384228179@163.com, 13550333680@163.com , tiamozyp@outlook.com

* Correspondence: gujianyi@163.com ; +86 135-9402-5638

Abstract: The study investigates the impact of different volume fractions of mixed steel fibers on the mechanical properties of self-compacting concrete. Preliminary tests were conducted on ordinary self-compacting concrete, single steel fiber concrete, and self-compacting concrete with 3% high-volume mixed steel fibers. The results show that high-volume mixed steel fiber self-compacting concrete specimens exhibit good bending performance, and the addition of steel fibers significantly inhibits the development of macro cracks, enhancing the concrete's resistance to cracking, effectively improving the brittleness of the concrete; when the fiber volume fraction is 1% 6mm+1% 13mm+1% 25mm, the compressive strength increased by 41.15%, and the flexural strength increased by 266.41%, and the elastic modulus reached 40.82MPa. Adding high-volume mixed steel fibers significantly improves the elastic modulus of self-compacting concrete, and the effect of mixed steel fibers is more pronounced.

Keywords: Self-compacting concrete; Blended steel fibers; Mechanical properties; Elastic modulus

0. Introduction

One typical building material is concrete. Researchers are looking at the performance-enhancing advantages of adding steel fibers to concrete to increase its mechanical qualities. Determining the impact of steel fibers on enhancing concrete's performance after adding a specific quantity to the material has long been a popular area of study. Concrete's ductility and toughness can be enhanced by the inclusion of randomly distributed steel fibers, which also improves the concrete's resistance to impact and cracking. Steel fibers can help increase the resilience of concrete and resistance to freeze-thaw[1-2].

When steel fiber content rises, concrete's workability suffers from a number of issues, including easier agglomeration and decreased workability. The amount of conventional steel fibers in concrete is restricted at low doses, and the distribution of steel fibers will be impacted by vibration during

construction. The flexural and tensile properties of concrete can only be slightly improved by steel fibers. In actual applications, the content of steel fibers is typically between 0.5% and 2% due to poor fiber dispersion in concrete[3].

Numerous studies have demonstrated that self-compacting concrete's mechanical qualities and durability can be successfully increased by incorporating the proper quantity of single steel fibers into the material. Steel fibers with lengths of 33 mm and volume fractions of 0.1%, 0.2%, and 0.3% were tested to see how they affected the compressive strength of self-compacting concrete by M. Akbari et al[4]. The findings show that as the volume fraction rises, so does the compressive strength.

Athiyamaan V[5] mixed 0.25%, 0.50%, and 0.75% of hook-end micro-steel fibers with an aspect ratio of 60 with self-compacting concrete. The findings demonstrated that adding steel fibers to self-compacting concrete increased its flexural strength, inhibited the growth of cracks, avoided brittle failure, and enhanced strain performance.

Researchers[6] have found that adding a specific amount of steel fibers to self-compacting concrete can increase its strength (Aghaee et al., 2016). For steel fibers, a dosage of 0.75% is ideal.

Researchers like Mardani-Aghabaglou [7] discovered that the compressive strength of self-compacting concrete dropped by 6.3% and 9.5%, respectively, when the steel fiber percentage increased to 0.75 and 1.0%. This decline aligns with the self-compacting theory. It has to do with the concrete losing its capacity to self-compact due to a decline in the workability of the concrete mixture itself, which lowers the effectiveness of self-compacting concrete.

For the purpose of to conduct comparative studies, researchers like Saba et al. [8] discovered that after adding 0~15% silica fume to self-compacting concrete, they added steel fibers with a volume content of 0~1% and an aspect ratio of 65~80. The test findings indicate that self-compacting concrete is significantly strengthened and toughened by steel fiber with an aspect ratio of 85 when the silica fume content is 15%, the volume content is 1%, and these conditions hold.

Researchers Sun et al. [9] discovered that the performance of self-compacting concrete is affected in a number of ways by the addition of steel fibers. They added steel fibers to self-compacting concrete in experimental trials at varying volumetric doses. The findings demonstrated that adding steel fibers by volume to self-compacting concrete will The tensile and flexural strengths of self-compacting concrete rise by 70% when the amount is increased from 0.5% to 1.25%, but the compressive strength reduces by 11%. It demonstrates that adding steel fiber greatly increases the tensile and flexural strength of self-compacting concrete while inhibiting its compressive strength.

Ma et al. [10] discovered that the compressive strength of self-compacting concrete could be raised by 19%–42% by adding steel fibers with a volume content of 0~2%, a length of 13 mm, and an aspect ratio of 65.

When choosing steel fibers for hybrid fiber-reinforced self-compacting concrete, two common varieties are considered. One is regular steel fiber, which has an aspect ratio of 40 to 65, a length that typically ranges from 15 to 35 mm, and a diameter that is mostly concentrated at 0.5 mm. Regular steel fiber is the other. It is a steel fiber that is extremely short and fine. Its shorter length makes it easier to mix uniformly than regular steel fiber and won't significantly impair concrete's performance. Different methods of mixing two kinds of steel fibers are used to enhance the mechanical characteristics of self-compacting concrete.

At present, most research focuses on improving the mechanical properties of self-compacting concrete after mixing two steel fibers with different aspect ratios. Burcu Akcay[11] et al. artificially studied the effects of steel fibers with different aspect ratios on the performance of self-compacting concrete, Add 1% straight steel fiber in volume fraction to self-compacting concrete (Length 6mm, diameter 0.15mm) and 0.5% hook-end steel fiber (length 30mm, diameter 0.55mm), The results show that these fibers can be dispersed evenly in self-compacting concrete without clumping, thus improving the toughness of the concrete. Deeb[12] et al. added 5% steel fiber (Length 6mm, diameter 0.16mm) and 1% steel fiber (Length 13mm, diameter 0.16mm) by volume to self-compacting concrete, experimental studies were conducted on the fluidity of concrete. The results prove that ultra-short ultra-fine steel fibers mixed with a higher content can produce self-compacting concrete with fluidity that meets the requirements. However, the article does not describe the enhanced effect of hybrid steel fibers on the properties of self-compacting concrete. Dimas[13-15] et al. used 12mm straight-end steel fiber (diameter is 0.18mm) with a volume content of 0.5% and 1.5% 35mm hook-end steel fiber (diameter 0.55mm) as reinforcing fibers. The mechanical properties such as fracture and bending resistance of self-compacting concrete were studied through compression, tension, and bending tests. Experimental results show that the normal serviceability limit state of self-compacting concrete has been improved.

Scholarly research based on the aforementioned data indicates that little is known about the hybrid impacts of three distinct aspect ratio steel fibers at this time. On the effect of hybrid reinforced self-

compacting concrete on performance, more investigation and analysis can be carried out. Drawing from prior studies, this investigation aimed to examine the effects of adding steel fibers with varying aspect ratios to self-compacting concrete at a volume content of 3% on the concrete's properties. To achieve self-compacting concrete, this work conducts experimental research on the fundamental mechanical properties of 3% hybrid steel fibers. The result is a high-content, self-compacting steel fiber concrete that performs exceptionally well throughout construction.

1. Materials and methods

1.1 Test materials and proportions

（1）Cement.The cement is P·O42.5 ordinary Portland cement produced by Chongqing Lafa base's dimensional cement formula. Its physical and mechanical properties are shown in Table 1.

Table1 Basic physical and mechanical properties of cement

Apparent density/（g • cm-3）	Specific surface area/(m2 • kg-1)	Sieve residual ratio(45μm sieve, by mass)/%	Water demand ratio for normal consistency (by mass)/%	Setting time/min		3d strength/MPa	
				Initial	Final	Compressive	Flexural
3.1	345	8.4	26.80	165	226	30.0	5.90

Formatted: Font: 10 pt, Bold
Formatted: Line spacing: single
Formatted Table
Formatted: Font: 10 pt, Bold
Formatted: Line spacing: single

（2）Fly ash. Fly ash is low calcium Class F fly ash produced and provided by Chongqing Hualuo Fly Ash Co., Ltd.

Table 2 Physical properties of fly ash

Apparent density/ g • cm-3）	Sieve residual ratio(45 μm sieve, by mass)/%	IL(by mass)/%	Water demand ratio(by mass)/%	Activity index/%	
				7d	28d
2300	0	5	95	83	95

Formatted: Font: 10 pt, Bold
Formatted: Line spacing: single
Formatted Table
Formatted: Font: 10 pt, Bold
Formatted: Line spacing: single
Formatted: Font: 10 pt
Formatted: Line spacing: single

（3）Aggregate. The fine aggregate is limestone machine-made sand with a fineness modulus of 3.2, a fine powder content of 3%, an apparent density of 2712kg/m³, and a bulk density of 1521 kg/m³, Coarse aggregate is 5~10mm crushed stone, with an apparent density of 2.78g/cm³ and a bulk density of 1.45g/cm³.

Table 3 Gradation of fine aggregate

Sieve diameter/mm	Sieve residual ratio(by mass)/%	Cumulative sieve residual ratio(by mass)/%	Di/mm
4.75	0	0	0
2.36	19.3	19.3	3.35
1.18	17.4	36.7	1.67
0.60	22.1	58.8	0.84
0.30	21.7	80.5	0.42

Formatted: Font: 10 pt, Bold
Formatted: Line spacing: single
Formatted Table

0.15	13.7	94.2	0.21
0.075	3.6	97.8	0.11
0	2.2	100	0.03

Table 4 Gradation of coarse aggregate

**Sieve diameter/mm**	**Sieve residual ratio(by mass)/%**	**Cumulative sieve residual ratio(by mass)/%**	**Di/mm**
16	0	0	0
13.2	0.27	0.27	14.53
9.5	0.40	0.67	11.20
4.75	0.33	1.00	6.74

Formatted: Font: 10 pt, Bold

Formatted: Line spacing: single

（4）Steel fiber. The main performance is shown in Table 5.

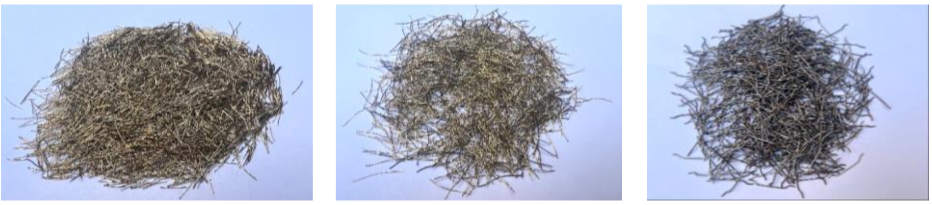

6mm 13mm 25mm

Figure 1 Steel fibers with different aspect ratios

Table 5 Types of steel fibers and main physical and mechanical properties

**Steel fiber types**	**Length /mm**	**Aspect ratio**	**Density（t/m³）**	**Elastic Modulus（GPa）**	**Tensile strength（MPa）**
Ordinary steel fiber (hook-end)	25	33	7.8	210	850
Ordinary steel fiber	13	65	7.8	240	＞850
Ultra-short ultra-fine steel fiber	6	35	7.8	240	＞850

Formatted: Font: 10 pt, Bold

Formatted Table

（5）Water-reducing agent. Water reducing agent (WR) is a high-efficiency polycarboxylate water-reducing agent mother liquor with a solid content (mass fraction) of 50% to 60% and should be diluted 6.5 times.

（6）Water. Using tap water from the laboratory of Chongqing Jiaotong University.

（7）Mix ratio selection. The mix ratio of this test was carried out based on the mix ratio determined in the previous test study. Table 6 records the mix ratio of steel fiber self-compacting concrete with different steel fiber volume content of 1m³.

Table 6 Mix ratio of steel fiber self-compacting concrete specimens (1m³/kg)

**Number**	**Cement（kg）**	**Water（kg）**	**Fly ash（kg）**	**Coarse aggregate（kg）**	**Fine aggregate（kg）**	**Water reducing agent（%）**	**Steel fiber content（%）**
1	1	0.37	0.43	1.37	1.06	0.16%	0
2	1	0.37	0.43	1.37	1.06	0.16%	3%6mm
3	1	0.37	0.43	1.37	1.06	0.16%	3%13mm

Formatted: Font: 10 pt, Bold

Formatted Table

4	1	0.37	0.43	1.37	1.06	0.16%	3%25mm
5	1	0.37	0.43	1.37	1.06	0.16%	1%6mm+1%13mm+1%25mm

(For the convenience of research, 3%6 represents 6mm steel fiber with a volume content of 3%, and other naming methods are the same)

Self-compacting steel fiber concrete should use a mandatory mixer. In this experiment, coarse aggregate, fine aggregate, and cement are first poured in, and then the steel fibers are slowly added in batches. The water mixed with the water-reducing agent is poured into the mixer. After the addition is completed, continue stirring for one minute. When there is a large amount of fiber or fiber, a fiber disperser can be used.

1.2 Experiment preparation

Before the test, the self-compacting concrete fluidity test and passing ability test were carried out [16]. The slump expansion diameter and J-ring expansion of self-compacting concrete with different volume dosages are shown in Figure 2 and Figure 3.

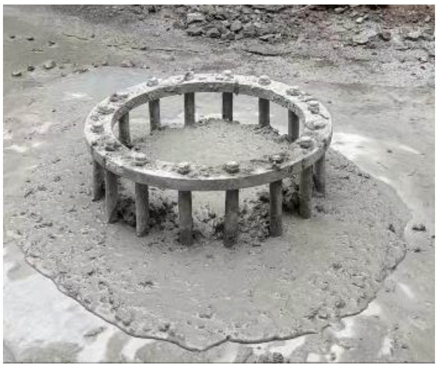

Figure 2 Slump expansion of steel fiber self-compacting concrete

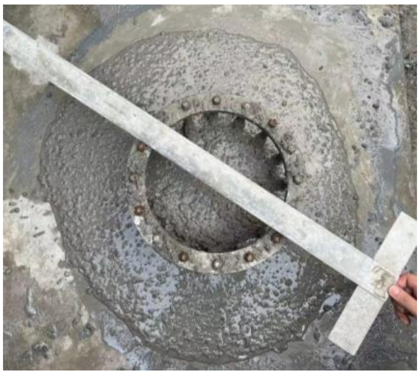

Figure 3 J-ring expansion of steel fiber self-compacting concrete

The experimental data are shown in Table 7, The experimental data are shown in Table 7, according to the "Technical Specification for Application of Self-Compacting Concrete" JGJ/T 283-2012, the standard for the slump flow of self-compacting concrete is 500mm to 800mm.

Table 7 Slump expansion diameter and J-ring expansion of steel fiber self-compacting concrete under different steel fiber content

Number	Length	Fiber content	slump propagation（mm）	J ring expansion（mm）
S-0	0	0	840	800
3%6	6mm	3%6mm	660	500

Formatted: Font: 10 pt, Bold
Formatted: Line spacing: single
Formatted Table

3%13	13mm	3%13mm	520	410
3%25	25mm	3%25mm	730	610
6+13+25	6mm+13mm+25mm	1%6mm+1%13mm+1%25mm	650	530

The target value for the slump flow of self-compacting concrete should not be less than 500mm; therefore, the self-compacting concrete with various steel fibers meets the construction requirements.

1.3 Experimental method

In this study, the mechanical properties of self-compacting concrete were characterized by compressive strength, flexural strength, and elastic modulus, so cube specimens and beam specimens were prepared for corresponding tests. This experiment conducted a preliminary experimental study on five groups of specimens: self-compacting concrete, single-content steel fiber concrete, and self-compacting concrete mixed with ordinary steel fiber and hook-end steel fiber at 3% content. Comparative analysis of the effect of steel fibers on the properties of self-compacting concrete. The number of test specimens for each group of solutions is 30 cube compressive strength test, flexural test, and bending and tensile test. The total number of specimens is 90. The size and quantity of specimens are summarized in Table 8.

Table 8 Dimensions and quantity of specimens

Test content	Specimen size（mm）	Number of test pieces	Number of samples
Compressive strength	100×100×100	6	30
Flexural strength	100×100×400	6	30
Elastic Modulus	100×100×400	6	30

Formatted Table

1.3.1 Cube compressive strength test

The primary indicators for evaluating the strength of concrete materials are compressive strength and flexural strength[17]. Use a cube test block with a size of 100mm×100mm×100mm, Prepare three identical specimens in each group, remove the mold after 24 hours of molding, After curing the specimen in the curing box for 28 days, and clean the surface of the specimen. Place the axis of the specimen perpendicularly to the center of the lower plate, and adjust the upper-pressure plate to be close to but not touching the upper surface of the test piece. Start the testing machine to preload. The loading speed during preloading is 0.5MPa/s, perform physical alignment adjustments and pressure stability adjustments. After the preloading is completed, the specimen is loaded at a constant speed of 2KN/s until the specimen is damaged, and then the load is slowly unloaded.

The compressive strength results of the test block should be calculated according to the following formula：

$$f_{cc} = \frac{F}{A} \quad (1.1)$$

In the formula：

f_{cc}—Steel fiber concrete cube compressive strength（MPa）；

F—Ultimate load（N）；

A—Average value of the upper and lower pressure areas of the test block（mm^2）。

1.3.2 Flexural strength test

According to the Chinese National Standard GB/T 50081—2002 "Standard for Testing Methods of Mechanical Properties of Ordinary Concrete." The size of the self-compacting concrete steel fiber test block is 100mm×100mm×400mm. The test was conducted on a hydraulic universal testing machine. The long axis of the test block is perpendicular to the support axis. Load at a constant speed of 50N/s until the test block is destroyed. After the specimen is cured in the curing box for 28 days, and the surface and inspected the surface of the specimen, Place the specimen on the testing machine with its long axis perpendicular to the support cylinder. The distance between the two cylindrical supports below is 0.3 meters, and constraints adopt fixed hinges and universal rolling hinge supports. Before starting the testing machine, first preload the test block with a loading force of 1kN. Check whether the test block is placed flat to ensure that the test is carried out in a standardized and safe manner. Before the initial failure of the test block occurs, the loading rate is 0.06MPa/s. Once cracks appear in the test block, the loading method is changed, displacement control is used, the load is loaded until the test piece is damaged, and then the load is slowly unloaded. The loading process is shown in Figure 4.

The flexural strength of the test block is expressed in MPa and is calculated according to the following formula:

$$f_r = \frac{3FL}{2bh^2} \quad (1.2)$$

In the formula:

f_r—Steel fiber concrete flexural strength, MPa,

P—Destruction load applied to the middle part of the specimen when breaking (N),

L—Support spacing (mm),

b—Specimen height (mm),

h—Specimen width (mm).

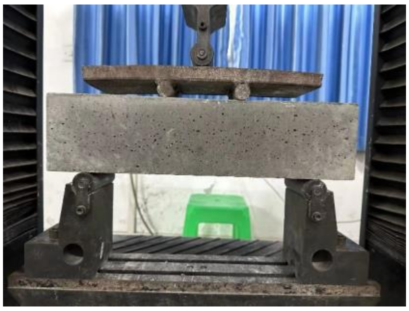

Figure 4 Flexural test loading of self-compacting steel fiber concrete

1.3.3 Elastic modulus test

According to GB/T 50081-2002 "Standard for Test Methods of Mechanical Properties of Normal Concrete", the specimen is a prism with edge lengths of 150mm × 150mm × 300mm. Attach the strain gauge to the specimen whose side hole diameter is less than 5mm and depth is less than 2mm. In order to consider the error factor of uneven distribution of aggregates, use a smear rod to stick to the strain gauge at the center of the left and right sides of the specimen, and the center lines of both sides need to coincide. After the smeared area is dry, place the specimen on the pressure-bearing platform according to the specifications and prepare to add load on the universal testing machine. Take 1/2 of the average value of the bending and tensile ultimate load as the load standard F0.5 for the bending and tensile elastic modulus test, and perform 5 loading and unloading cycles, starting from 10KN and loading at a speed of 0.2mm/min.

The flexural and tensile elastic modulus of concrete should be calculated as follows:

$$E_f = \frac{23L^3(F_{0.5}-F_0)}{1296J|\Delta_{0.5}-\Delta_0|} \qquad (1.4)$$

In the formula:

E_f—Concrete bending and tensile elastic modulus (MPa),

$F_{0.5}$、F_0—Final load and initial load (N),

$\Delta_{0.5}$、Δ_0—Corresponding to the dial indicator reading of $F_{0.5}$ and F_0 (mm);

L—Distance between specimen supports (L=450mm);

J—Moment of inertia of specimen section, $J = \frac{1}{12}bh^3$ (mm4).

2. Results and analysis

2.1 Cube compressive strength

Figure 5 shows the compression failure modes of ordinary concrete, single dosage, and hybrid steel fiber concrete cubes.

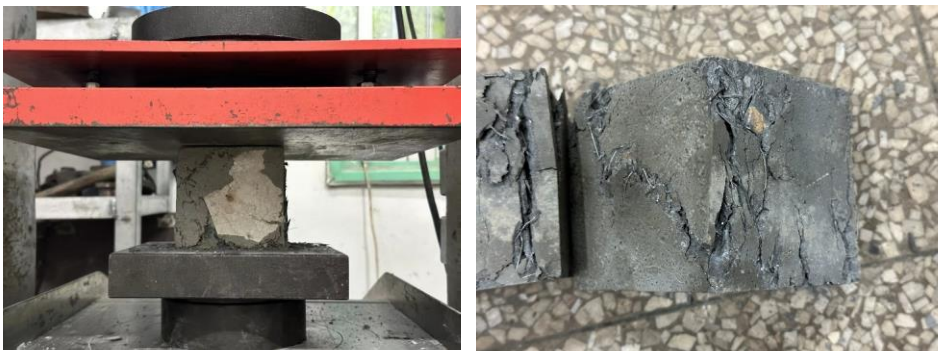

Figure 5 Compression failure pattern of concrete cube specimens

According to the compression failure pattern of the concrete specimen in Figure 2-1, it can be seen that when the self-compacting concrete specimen is mixed with steel fibers, its peeling speed slows down under compressive load, the time it continues to bear pressure after peeling increases, and the peeling is more uniform.

According to the test, the data shown in Table 9 is obtained. The data in the table shows that the addition of steel fibers can effectively improve the compressive strength of self-compacting concrete, Compared with ordinary self-compacting concrete, when the fiber content is 3%, the increase in the compressive strength of concrete with steel fibers with different aspect ratios is not much different, Among them, the addition of 13mm steel fiber greatly improves the compressive strength of self-compacting concrete. By mixing three types of steel fibers into self-compacting concrete, the compressive strength of the concrete has been significantly improved. Compared with ordinary self-compacting concrete, the compressive strength has increased by 41.15%. At the same time, when the volume fraction of steel fibers is the same, the compressive strength of hybrid steel fiber concrete is increased by about 20% compared with steel fiber alone. But overall, the addition of steel fibers has a limited effect on enhancing the compressive strength of self-compacting concrete.

Table 9 Cube compressive strength test results (28d, MPa)

Number	Fiber content	Compressive strength (MPa)	Improvement
S-0	0	73.65	—
3%6	3%6mm	82.56	12.09%
3%13	3%13mm	85.28	15.79%
3%25	3%25mm	83.26	13.05%

Formatted: Font: 10 pt, Bold, Font color: Auto
Formatted: Line spacing: single
Formatted Table

6+13+25	1%6mm+1%13mm+1%25mm	103.96	41.15%

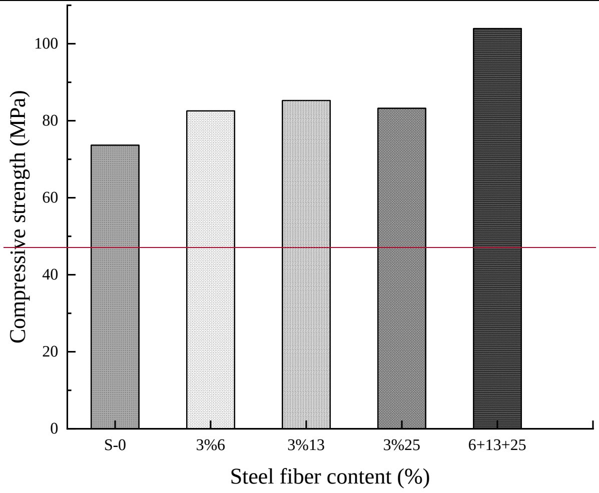

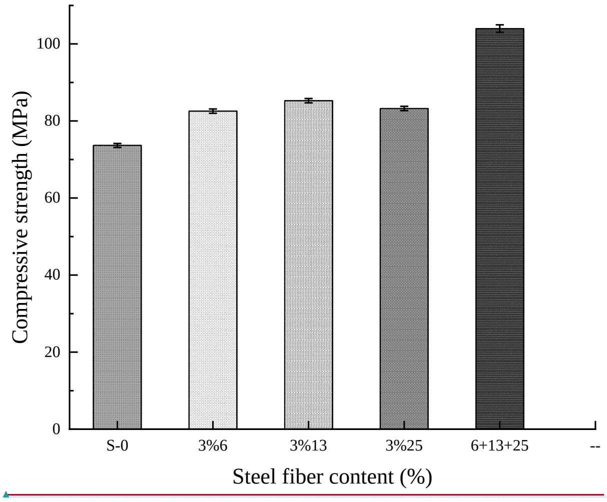

Field Code Changed

Figure 6 The relationship between the compressive strength value of self-compacting steel fiber concrete cube and the volume content of steel fiber

2.2 Flexural strength

Figure 7 shows the flexural failure morphology of ordinary concrete, single dosage, and hybrid steel fiber concrete cubes.

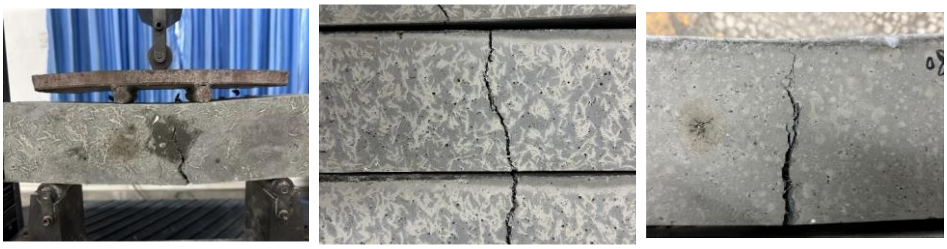

Figure 7 Concrete flexural failure pattern

It can be seen from the failure shape that under the ultimate load, cracks soon appeared at the edge of the tensile zone at the bottom of the concrete specimen without adding steel fibers. Under the influence of stress concentration and tip effect, the crack immediately expanded to the top, and a clear crack appeared. At the same time, several concrete blocks fell off in the tension area, accompanied by a crisp and loud breaking sound, plain concrete blocks quickly failed and stopped working. The flexural concrete test block with steel fiber added will have a slower time of cracking than the plain concrete test block. Under the action of concentrated force, a main crack first appears in the bottom tension zone. As the load increases, the main crack gradually expands from the bottom of the tension zone to the top of the beam until the specimen is completely cracked. The flexural strength obtained through the test is shown in Table 10.

Table 10 Flexural strength test results (28d, MPa)

Number	Fiber content	Flexural strength (MPa)	Improvement
S-0	0	3.93	—
3%6	3%6mm	8.41	113.95%
3%13	3%13mm	14.96	280.66%
3%25	3%25mm	12.24	211.45%
6+13+25	1%6mm+1%13mm+1%25mm	14.47	268.19%

As can be seen from Figure 8, steel fibers can effectively improve the flexural strength of self-compacting concrete. Compared with ordinary self-compacting concrete, the flexural strength of self-compacting concrete mixed with fibers has a greater increase. When the volume content is 3%, the 13mm steel fiber has the most obvious effect on enhancing the flexural strength of self-compacting concrete, while the addition of ultra-short and ultra-fine 6mm steel fibers has no significant effect on the flexural strength of self-compacting concrete. This shows that as the aspect ratio of steel fibers increases, the flexural strength of self-compacting concrete continues to increase. After mixing three steel fibers with different aspect ratios, mixing 1%6mm+1%13mm+1%25mm into self-compacting concrete, it can be seen that the reinforcing effect of mixed fibers on self-compacting concrete is not as good as the reinforcing effect of 13mm steel fiber alone. Analyze the reasons: Steel fibers with

Formatted: Font: Bold

Formatted Table

different aspect ratios within the concrete are not easily dispersed fully and evenly, and the agglomeration of the fibers with cement slurry or aggregate increases the internal pores of the specimen, resulting in a limitation in the enhancement effect of mixed fibers on the flexural strength of self-compacting concrete.

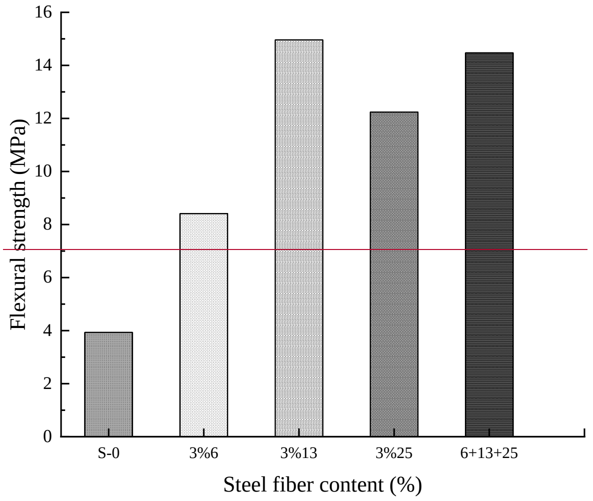

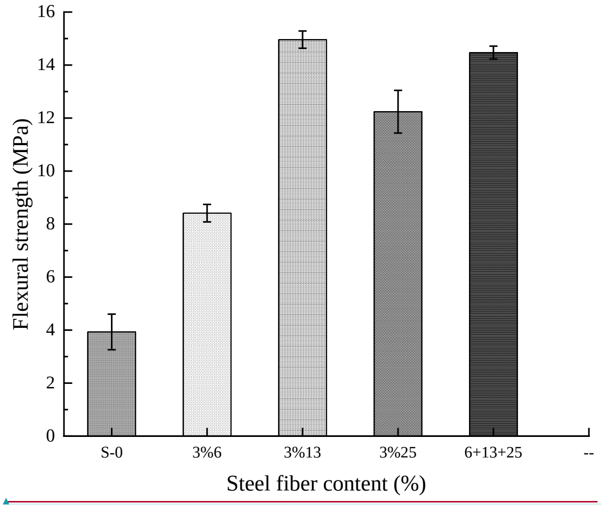

Figure 8 The relationship between the flexural and tensile elastic modulus of self-compacting steel fiber concrete and the volumetric content of steel fiber

2.3 Elastic Modulus

Field Code Changed

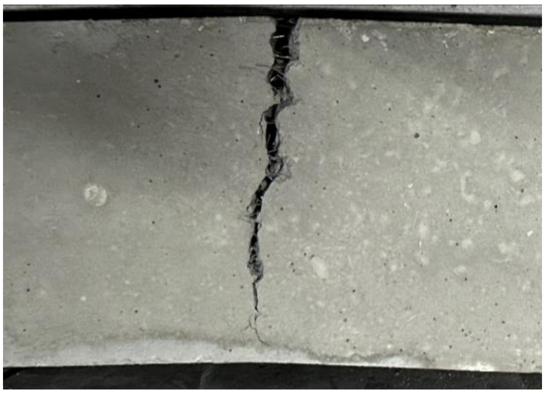

Figure 9 Elastic Modulus test loading of self-compacting steel fiber concrete

According to the test results, it can be seen that ordinary self-compacting concrete specimens have very long cracks after being loaded. Basically running through the entire rectangular parallelepiped, the crack develops from top to bottom from the upper platform, and the width of the crack is very wide when it is close to the upper platform, and is narrower near the lower platform. The cracks in the mixed steel fiber specimens have a narrower width, develop slower, and can withstand the load for a longer time. The elastic modulus obtained through the test is shown in Table 11.

Table 11 Elastic modulus test results (28d, MPa)

Number	Initial load $F_0(kN)$	Test piece pressure area $A(mm^2)$	Average elastic modulus $E_c(MPa)$
S-0	10.0	10000	26490
3%6	10.0	10000	34880
3%13	10.0	10000	35230
3%25	10.0	10000	32820
6+13+25	10.0	10000	40820

Formatted: Font: 10 pt, Bold
Formatted: Font: 10 pt, Bold
Formatted: Line spacing: single
Formatted Table
Formatted: Font: 10 pt, Bold
Formatted: Font: 10 pt, Bold

It can be seen from Figure 10 that when steel fibers are added, the elastic modulus of self-compacting concrete is significantly improved. At the same dosage, steel fibers with lengths of 6 mm and 13 mm improve the elastic modulus of self-compacting concrete more than the addition of 25 mm steel fibers, indicating that the performance improvement of self-compacting concrete by hook-end steel fibers is weak. It can be clearly seen from the figure that hybrid steel fibers have a more

obvious increasing trend in the elastic modulus of self-compacting concrete. This shows that the incorporation of hybrid steel fibers has a certain effect on improving the elastic modulus, but generally speaking, steel fibers have little effect on the elastic modulus.

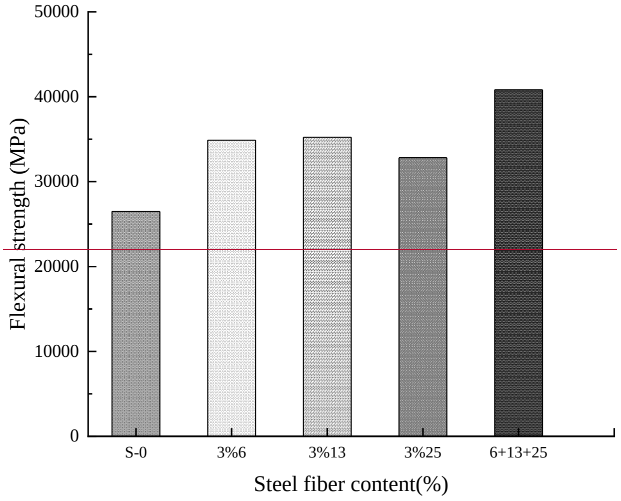

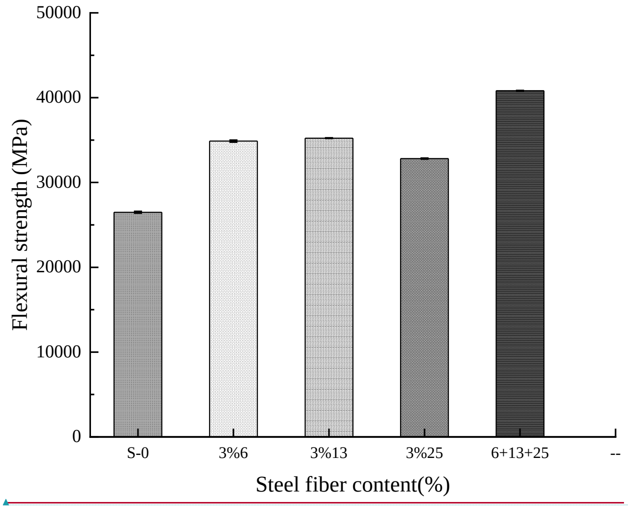

Field Code Changed

Figure 9 The relationship between the elastic modulus of self-compacting steel fiber concrete and the volume content of steel fiber

2.4 Tension-compression ratio

In order to study the impact of steel fiber on the strength of concrete, this article further analyzed the test data conducted previously to obtain the tension-to-compression ratio of the specimen and plotted it with the steel fiber content as the X-axis and the tension-to-compression ratio as the Y-axis.

As shown in Figure 11, The Y-axis data is the ratio of the flexural strength of the specimen to the compressive strength of the specimen.

Field Code Changed

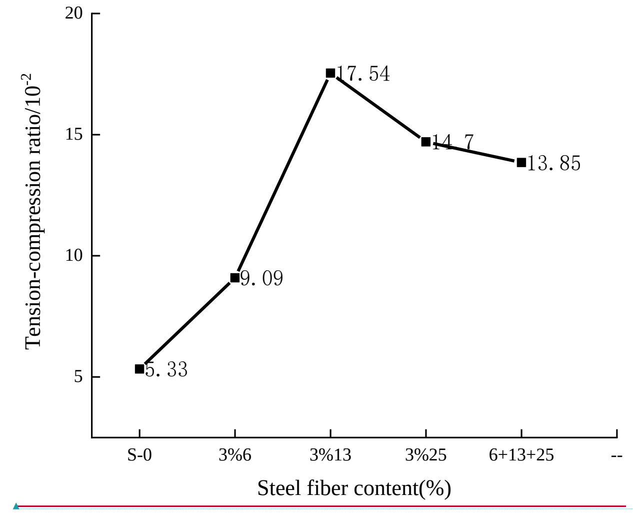

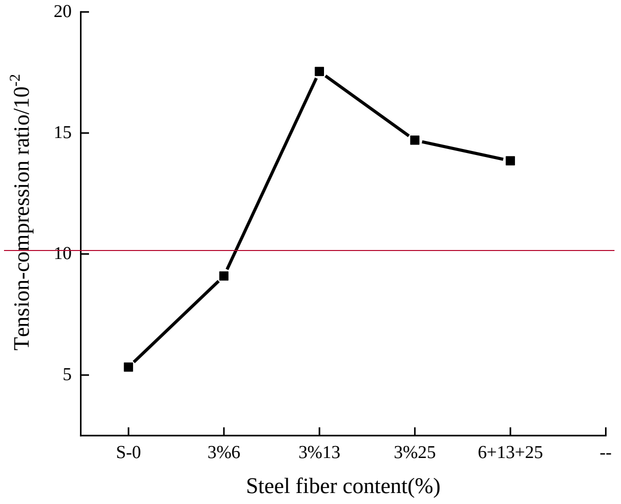

Figure 11 The relationship between the tension-compression ratio of self-compacting steel fiber concrete and the volume content of steel fiber

The influences of steel fibers with different lengths and contents on the tension-compression ratio of concrete are diverse. Among them, the 13mm steel fibers with a volume content of 3% can enhance the tension-compression ratio of concrete to the greatest extent. While the effect of mixing steel fibers of multiple lengths is relatively weaker, it is still better than the situation without steel fibers and some cases of single-length steel fibers. Consequently, steel fibers can effectively improve

the tension-compression ratio of self-compacting concrete.

3. Discussion

3.1 Optimal dosage

According to the compression, flexural, and elastic modulus test results, it can be seen that the addition of steel fibers can effectively improve the mechanical properties of self-compacting concrete. Since the flexural strength of 13mm steel fiber alone increases significantly, it is difficult to determine the specific mixing ratio that will have the most obvious improvement effect on self-compacting concrete.

In order to find the optimal blending ratio, data normalization is used to narrow the selection criteria to between 0 and 1. By accumulating the regularization criterion values, the comprehensive impact of steel fibers on self-compacting concrete is determined.

The data normalization formula is as follows,

$$D_n = \frac{D-D_{min}}{D_{max}-D_{min}} \quad (3\text{-}1)$$

In the formula:

D--- the original value of the test data,

D_{max} --- the maximum value of the test data,

D_{min} --- the minimum value of the test data,

D_n ---the normalized value of the test data.

In this study, the experimental data obtained above were imported into the Origin software, and the comprehensive impact of steel fibers on self-compacting concrete was obtained through normalized calculations, as shown in Figure 12. It can be seen from the normalized data that when the fiber content is 1%6mm+1%13mm+1%25mm, the improvement effect on the mechanical properties of self-compacting concrete is the best. It can be seen that hybrid steel fibers can effectively inhibit the development of concrete cracks and improve the cracking resistance of self-compacting concrete.

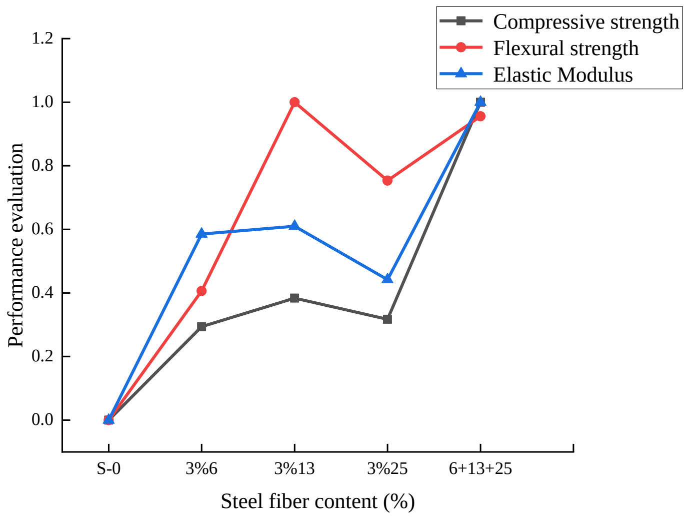

Figure 12 Comprehensive effect of fiber content on self-compacting concrete

3.2 Comparison

To prove the reliability of this study, this article selects three similar studies from previous years for comparative analysis. In 2019, researchers such as Alabduljabbar [19] added steel fibers with volume fractions of 0, 1%, 1.5%, and 2% to self-compacting concrete respectively. Research results show that when the volume content is 2%, the compressive strength and tensile strength of steel fiber reinforced self-compacting concrete reach the peak. Ghorbani (2020)[20] added steel fibers with a volumetric content of 0~1.65% to self-compacting concrete for research. The results showed that the performance of self-compacting concrete is optimal when the content is 1.65%. Abbas (2021)[21] also added steel fibers with a volume content of 0~2% in self-compacting concrete, the test results show that when the dosage is 2%, the self-compacting concrete has the highest strength. Based on the above three test results, this study comparatively analyzes the reinforcing effects of steel fibers at different dosages.

Table 12 Comparison of experimental data

**Refs.**	**Steel fiber percentages(%)**	**Days**	**Optimum(%)**	**Compression strength (MPa)**	**Flexure strength (MPa)**
Alabduljabbar et al.,(2019)	0~2	28	2	+17.2%	+1.96%
Ghorbani et al.,(2020)	0~1.65	28	1.65	-1.51%	+28.57%
Abbas et al.,(2021)	0~2	28	2	+40.0%	-
This Research	0~3	28	3	+41.15%	268.19%

Formatted: Font: Bold

Formatted Table

It can be seen from the growth data listed in Table 12 that different steel fiber content has different reinforcing effects on self-compacting concrete. Compared with other experimental research results, the mechanical properties of steel fiber reinforced self-compacting concrete obtained in this study have obvious hybrid effects. This study explains the reinforcement mechanism of self-compacting concrete by hybrid steel fibers with different aspect ratios. It can be seen from the data in the table that this research shows strong performance advantages in both compressive strength and flexural strength. Higher compressive strength means that the material is less likely to be damaged when subjected to pressure and can be applied to scenarios with higher requirements for bearing capacity. The extremely high flexural strength indicates that the material has better toughness and stability when subjected to bending forces, and is suitable for structures or components that need to resist bending deformation. This makes the research results potentially have wider applicability and competitiveness in practical applications.

4. Conclusion

（1）When mixed steel fibers with a volume content of 1% 6mm + 1% 13mm + 1% 25mm are added to the concrete, the compressive strength of self-compacting concrete increases by 41.15%; the flexural strength increases by 268.19% respectively. Therefore, the compressive and flexural strength of self-compacting concrete can be improved by incorporating hybrid steel fibers.

（2）Steel fibers can significantly improve the damage state of concrete. When a steel fiber concrete specimen fails, the cracks usually do not penetrate the entire specimen like ordinary concrete. The test block generally does not break into two pieces and has an obvious initial cracking load. The test block generally cracks without breaking, and its failure shows a certain ductility.

（3）When steel fibers are added to the concrete, the test block shows obvious ductility during the loading process, which is manifested as continuous cracking of the test piece.

It can be seen that the basic mechanical properties of the self-compacting hybrid steel fiber concrete obtained by mixing three different aspect ratio steel fibers meet the requirements of self-compacting concrete as the fiber volume content increases. It not only achieves self-compacting properties that are difficult to achieve at high dosages, but also meets the workability of the mixture and complies with national standards. Secondly, after multiple experimental tests, the self-compacting hybrid steel fiber concrete test block has good compressive and flexural properties. Moreover, steel fibers can significantly inhibit the development of macro cracks, making concrete more resistant to

cracking.

References:

[1] Zhino A. Shawais,Khamees N. Abdulhaleem,Saleh H. Ahmed,Hussein M. Hamada,Van R. Mohammed.Influence of Recycled Coarse Aggregate and Steel Fiber on the Workability and Strength of Self-Compacting Concrete[J].IOP Conference Series: Earth and Environmental Science,2024,1374(1):012084-012084.

[2] Nabeel A.Farhan,M.Neaz Sheikh,Muhammad N.S.Hadi.Effect of Steel Fiber on Engineering Properties of Geopolymer Concrete[J].ACI Material Journal,2020.5.

[3] Alberto José Palacio,Bárbara Belén Raggiotti,Viviana Carolina Rougier.Development and experimental verification of a design method for steel fibre-reinforced high strength lightweight self-compacting concrete[J].Construction and Building Materials,2024,453139012-139012.

[4] Mohamed M. Al Rifai,Karol S. Sikora,Muhammad N.S. Hadi.Effect of micro steel fibers volume fraction on behavior of high-strength self-compacting concrete[J].Construction and Building Materials,2024,450138709-138709.

[5] Athiyamaan V, G. Mohan Ganesh1. Analysis of the Alignment of Micro-Steel Fibers in Admixture-Based Self-Compacting Concrete (MSFR-SCC) using NDT and Evaluation of Its Effect on the Modulus of Rupture [J]. International Journal of Technology. 10(1): 5-15 2019.

[6] Aghaee, K., Yazdi, M. A., & Tsavdaridis, K. D. Investigation into the mechanical properties of structural lightweight concrete reinforced with waste steel wires. Magazine of Concrete Research, 67(4), 197–205.2015.

[7] Mardani-Aghabaglou, A., Tuyan, M., Yılmaz, G., Arıöz, Ö., & Ramyar, K. Effect of different types of superplasticizer on fresh, rheological and strength properties of self-consolidating concrete. Construction and Building Materials, 47, 1020–1025.2013.

[8] Saba, A. M., Khan, A. H., Akhtar, M. N., Khan, N. A., Koloor, S. S. R., Petru, M.,& Radwan, N. Strength and flexural behavior of steel fber and silica fume incorporated self-compacting concrete. Journal of Materials Research and Technology, 12,1380-1390.2021.

[9] Sun, L., Wenju, Hu., Yuan,Y, Cao, X., & Lei, Bo. Dynamic performance of the shading-type building-integrated photovoltaic claddings. Procedia Engineering, 121,930-937.2015.

[10] Ma, H. L., Cui, C., Li, X., & Hu, S. L. Study on mechanical properties of steel fiber reinforced autoclaved lightweight shell-aggregate concrete.Materials and Design, 1980–2015(52), 565–571.2013.

[11] Akcay,Burcu;Tasdemir, Mehmet Ali.Mechanical behaviour and fibre dispersion of hybrid steel fibre reinforced self-compacting concrete.[J].Construction & Building Materials.2012,Vol.28(No.1)：287-293.

[12] Deeb R.Development of self-compacting high and ultra high performance concretes with and without steel fibres[J].Cement&Concrete Composites,2012,34:185-190.

[13] Dimas Alan Strauss Rambo,Flávio de Andrade Silva,Romildo Dias Toledo Filho.Effect of steel fiber hybridization on the fracture behavior of self-consolidating concretes[J] Cement & Concrete Composites.2014.

[14] Dimas Alan Strauss Rambo,Flávio de Andrade Silva,Romildo Dias Toledo Filho.Flexural behavior of hybrid steel fiber reinforced self-consolidating concretes[J] Civil Engineering. 67(1), 27-32, jan. mar. 2014.

[15] Dimas Alan Strauss Rambo,Flávio de Andrade Silva,Romildo Dias Toledo Filho.Mechanical behavior of hybrid steel-fiber self-consolidating concrete: Materials and structural aspects[J] Materials and Design.2014.

[16] ASTM C 1621. Standard test method for passing ability of self-compacting concrete by J ring.[S] ASTM Standards; 2006.

[17] Iqbal, S., Ali, A., Holschemacher, K., & Bier, T. A. Effect of change in micro steel fiber content on properties of high strength steel fiber reinforced lightweight self-compacting concrete (HSLSCC). Procedia Engineering, 122, 88-94, 2023.

[18] RILEM 50-FMC. Determination of the fracture energy of mortar and concrete by means of three-point bend tests on notched beams.[J] Materials and Structures 1985; 18(4):285–90.

[19] Alabduljabbar, H., Alyousef, R., Alrshoudi, F., Alaskar, A., Fathi, A., & Mohamed,A. M. Mechanical effect of steel fiber on the cement replacement materials of self-compacting concrete. Fibers, 7(4), 36.2019.

[20] Ghorbani, S., Sharif, S., Rokhsarpour, H., Shoja, S., Gholizadeh, M., Rahmatabad,M. A. D., & de Brito, J. Effect of magnetized mixing water on the fresh and hardened state properties of steel fibre reinforced self-com- pacting concrete. Construction and Building Materials,248, 118660.2020.

[21] Abbas, S. A., Ali, 1. F., & Abdulridha, A.A. Behavior and strength of steel fiber reinforced self-compacting concrete columns wrapped by carbon fiber reinforced polymers strips. International Journal of Engineering, 34(2),382-392.2021.

The authors would like to thank the reviewers for their suggestions. All tables have been revised, and all figures have been redesigned, with error bars or numerical values added to the corresponding data as indicated.
