# Supplementary material for: Research on the mechanical properties of hybrid Steel Fiber-Reinforced Self-Compacting Concrete based on the DIC method
Source: PLoS One. 2025 May 15;20(5):e0322450. doi: 10.1371/journal.pone.0322450 (PMC12080768; doi:10.1371/journal.pone.0322450)
Supplement: Attachment — Submitted filename: Response to reviewers.docx [file pone.0322450.s002.docx]

Reviewer #1: Complete review of English – adjust verb tenses.

Correct the font numbering in the text, placing it in ascending order.

The introduction must include the most current references, preferably from the last 5 years.
Cite the standards for all tests performed in this study.
Table 4 Gradation of coarse aggregate - to correct
Table 7 – cite the limits of self-compacting concrete standards for each measured property and classify your concretes
Tables 9, 10, 11 – insert standard deviation, variance
Line 172 – “and shut down the fuel tank” - I don’t understand?
Correct equation 1.2
Regarding the Elastic modulus test: Specify the size of the test piece and the standard used to perform the test. Show a photo of the execution of this test.
Include in the article a statistical analysis of the results.

Reviewer #2: Make a careful review of the (tenses) verbs and verb agreements in the entire article.

Adjusted.
Correct the numbering of bibliographic references cited in the Article in ascending order – [1], [2], [3], etc.
Mention all standards used in all tests carried out in this research.

Table 7 – mention the limits of self-compacting concrete standards for each measured property and classify the traits of your concrete.
Tables 9, 10, 11 and 12 – and corresponding Figures 8, 9, 10 and 11 – insert all values of standard deviation, variance.

Correct equation (1.2) and the line symbol line 185 – ff

Cite the reference and norm of equation (1-4).

281 2.4 Tension-compression ratio

Present three current bibliographical references (last five years) that corroborate this methodology to evaluate the impacts of steel fiber or steel fibers on the strength of concrete results.

Cite the reference and norm of equation (3-1).

Prepare a statistical analysis of the results obtained.

Thank you for pointing this out. I agree with these comments. The tenses in the whole article have been carefully examined. The reference numbers cited in the article have been corrected and arranged in ascending order. The standards used in all the tests in the article have been clearly marked. Regarding Table 7, the limits of each measurement attribute in the self-compacting concrete standard have been mentioned in the article. Since there is only one set of performance parameters under different mixtures in the article, the calculation of standard deviation and variance is not possible. The mistakes within the formulas have been fixed, the bibliographic information has been renewed, and the outcomes of the article have been distinctly stated.
