# Supplementary material for: Research on the mechanical properties of hybrid Steel Fiber-Reinforced Self-Compacting Concrete based on the DIC method
Source: PLoS One. 2025 May 15;20(5):e0322450. doi: 10.1371/journal.pone.0322450 (PMC12080768; doi:10.1371/journal.pone.0322450)
Supplement: Attachment — Submitted filename: Response to reviewers-V.docx [file pone.0322450.s004.docx]

The authors would like to thank the reviewers for their suggestions. All tables have been revised, and all figures have been redesigned, with error bars or numerical values added to the corresponding data as indicated.
