# Supplementary material for: Research on the mechanical properties of hybrid Steel Fiber-Reinforced Self-Compacting Concrete based on the DIC method
Source: PLoS One. 2025 May 15;20(5):e0322450. doi: 10.1371/journal.pone.0322450 (PMC12080768; doi:10.1371/journal.pone.0322450)
Supplement: Attachment — Submitted filename: Response_to_reviewers-V_auresp_3.docx [file pone.0322450.s005.docx]

**Dear Reviewer,**

Thank you very much for your valuable comments and suggestions, which have helped us significantly improve the quality of our manuscript. Below, we provide a point-by-point response to your comments:

**Comment 1: Highlight the difference in this study and its scientific advance.
Response:**

We sincerely appreciate your suggestion. In the revised manuscript, we have emphasized the unique aspects of our study in the Introduction and Discussion sections. Specifically, our research focuses on the synergistic effects of hybrid steel fibers (1%6mm + 1%13mm + 1%25mm) on the mechanical properties of self-compacting concrete, which has not been systematically investigated in previous studies. Additionally, we employed the Digital Image Correlation (DIC) method to capture the full-field strain evolution and crack propagation, providing new insights into the macro- and micro-crack dynamics of hybrid fiber-reinforced concrete.

**Comment 2: The introduction needs more information with specific data and greater depth.
Response:**

We have revised the Introduction section to include more specific data and a deeper discussion of the current state of research. We have added recent references on steel fiber-reinforced concrete and highlighted the limitations of existing studies, such as the lack of systematic investigation into hybrid steel fibers.

**Comment 3: Highlight the importance of the manuscript with schematic figures or numerical simulations.**
**Response:**

Thank you for this suggestion. In the revised manuscript, the full-field strain evolution during specimen failure was obtained using the DIC method, and the dynamic evolution characteristics of macro- and micro-cracks on the specimen surface were analyzed, demonstrating the mechanical behavior of concrete under different fiber contents.

**Comment 4: Improve the English writing and logical flow.
Response:**

We have carefully revised the manuscript to improve the English language and ensure logical coherence.

**Comment 5: Perform statistical analysis to assess the results.**
**Response:**
We have conducted a statistical analysis to evaluate the significance of the differences in mechanical properties among the tested concrete samples. The results of this analysis are presented in the revised manuscript, and we have discussed their implications in the Results and Discussion sections.

Once again, we thank you for your constructive feedback, which has greatly improved the quality of our work. We hope that the revised manuscript meets your expectations.

Sincerely,

Jianyi Gu
